# Manganese modulates hepatocellular carcinoma cytotoxicity and doxorubicin sensitivity in a dose dependent manner

**DOI:** 10.3389/fonc.2026.1715702

**Published:** 2026-02-13

**Authors:** Hao-lun Wang, Qian-qing Fan, Ting Tang, Zi-yun He, Yu-an Xie, Zhi-hui Liu

**Affiliations:** 1 Department of Medical Oncology, Guangxi Medical University Cancer Hospital, Nanning, China; 2 Graduate School of Guangxi University of Traditional Chinese Medicine, Guangxi Key Laboratory of Reproductive Health & Birth Defects Prevention, Nanning, China; 3 Guangxi Key Laboratory of Reproductive Health and Birth Defects Prevention, Guangxi University of Traditional Chinese Medicine, Nanning, China; 4 Day-care Unit, Guangxi Medical University Cancer Hospital, Nanning, China

**Keywords:** cytotoxicity, dual effect, hepatocellular carcinoma, manganese, transcriptome sequencing

## Abstract

**Introduction:**

Hepatocellular carcinoma (HCC) is a leading cause of cancer-related mortality globally, characterized by decreased manganese (Mn) levels in tumor tissues compared to noncancerous liver tissues. Although manganese’s role in enhancing immune responses and inhibiting tumor growth has been noted, its specific contribution to HCC development and its influence on drug sensitivity are not well defined.

**Methods:**

We assessed the viability of HCC cells treated with various concentrations of MnCl_2_ using assays such as CCK-8, EdU staining, and flow cytometry. These assays revealed that MnCl_2_ at different concentrations could reduce doxorubicin sensitivity or induce cytotoxicity. Subsequently, transcriptome sequencing was employed to identify differentially expressed genes and those playing critical roles. The potential molecular mechanisms were investigated through functional enrichment analysis, and key genes were validated using Western blot (WB) analysis.

**Results:**

Our study found that low MnCl_2_ concentrations increased AKT pathway phosphorylation, enhancing doxorubicin resistance, while high MnCl_2_ concentrations activated the P53 pathway and immune response, downregulating mitosis and the MYC pathway.

**Discussion:**

This research elucidates the impact of Mn²^+^ concentration on HCC cell behavior, offering a theoretical basis for Mn²^+^’s potential use in HCC treatment. The findings contribute to a deeper understanding of Mn²^+^’s role in HCC and may inform future therapeutic strategies.

## 1 Introduction

Manganese is closely related to various physiological functions of the human body. In previous studies, manganese has been shown to be an important factor for the maintenance of many bodily functions, such as protein metabolism, cholesterol synthesis, bone development, aging, peroxidation, and the immune response (1, 2). Disruption of the manganese balance may lead to various diseases. The neurotoxicity of manganese causes neurological disorders, such as Parkinson’s disease-like symptoms, Alzheimer’s disease, Huntington’s disease and prion disease (3).

Therefore, the effect of manganese on hepatocellular carcinoma has also received extensive attention. Among them, Liu Zhihui and Chen Xiubing et al. reported that the manganese content of liver tumor tissues was lower than that of adjacent noncancerous tissues and normal tissues. Liu Zhihui also reported that the manganese content in hepatocellular carcinoma was related to chemotherapy sensitivity (4, 5). These findings indicate that manganese plays a role in the occurrence and development of hepatocellular carcinoma. Primary liver cancer (hepatocellular carcinoma) remains one of the leading malignancies in terms of incidence and mortality worldwide (6). Among patients with advanced HCC, the primary treatment modalities include molecular targeted drug therapy, immunotherapy, chemotherapy, and Chinese herbal medicine (7–9). However, traditional treatments still cannot meet clinical needs. Therefore, exploring the mechanism of tumorigenesis and new treatment models is still urgently needed in HCC. Actually, exploring the role of manganese in HCC may be a breakthrough point. Previous studies have shown that manganese levels in brain tissue have a biphasic effect on the nervous system (10, 11). However, the role of different concentrations of manganese and the mechanism of action of manganese in hepatoma cells are still unclear, and its effect on the transcriptome has not been clarified. Therefore, our study aimed to explore the role of different MnCl_2_ concentrations in hepatocellular carcinoma and to elucidate the underlying molecular mechanisms involved in the effects of MnCl_2_.

Recent studies have even shown the great potential of manganese due to its role in immune promotion and for its antitumor effects (12, 13) Notably, however, some studies have reported negative effects of manganese on tumors. For example, manganese can promote the metastasis and invasiveness of lung cancer cells and can induce resistance to chemotherapy drugs (4, 14).

Many studies have confirmed the feasibility of explaining biological changes and predicting experimental effects at the transcriptional level, and bioinformatics technology is an effective way to explore the mechanism of specific molecular effects (15).

The Huh7 cell line is a highly differentiated liver cancer cell line that can effectively simulate the biological characteristics of HCC cells. HepG2.2.15 is a hepatoblastoma cell line that can stably replicate HBV. Due to the close association between the pathogenesis of HCC and the expression and replication levels of HBV virus genes, the development of drugs for HBV-related liver cancer is particularly important. Therefore, we chose these two cell lines to explore the effects of different concentrations of MnCl_2_. In addition, we performed a series of bioinformatics analyses on the transcriptome data of MnCl_2_-treated HCC cells and verified the changes in the pathways of these cells through WB analysis. This study aimed to investigate the toxicity and protective effects of MnCl_2_ on liver cancer cells, analyze the potential impact of different MnCl_2_ doses on transcriptional changes in liver cancer cells, and explore the regulatory effects of different doses of MnCl_2_ on HCC pathways.

This study contributes to the understanding of MnCl_2_-induced cell death, its protective functions in HCC and the underlying molecular mechanisms involved. This study also provides a reference for exploring the possible effects of low doses of MnCl_2_ on the HCC phenotype.

## 2 Methods

### 2.1 Cell culture and processing

The human hepatocellular carcinoma cell line Huh7 was obtained from the Cell Bank of the Chinese Academy of Sciences (Shanghai, China) under the product catalog number SCSP-526. The HepG2.2.15 cell line was purchased from Shanghai iCell Bioscience, Inc. (Catalog Number: iCell-h093). Both cell lines were cultured and maintained in a humidified incubator at 37°C with 5% CO_2_.

Huh7 cells were cultured in Dulbecco’s Modified Eagle Medium (DMEM). The complete growth medium for Huh7 cells was prepared by supplementing DMEM with 10% (v/v) heat-inactivated fetal bovine serum (FBS; VivaCell Biosciences, catalog #C04001-500) and 1% (v/v) penicillin-streptomycin solution (Solarbio, catalog #P1400; final concentration: 100 U/mL penicillin and 100 µg/mL streptomycin). HepG2.2.15 cells were cultured using the specialized complete MEM medium provided by Shanghai iCell Bioscience, Inc. (catalog #iCell-h093-001b), which is formulated specifically for this cell line. The culture medium for both cell lines was refreshed every 2–3 days. Cells were passaged at approximately 80-90% confluence using 0.25% trypsin-EDTA solution.

For the experimental treatments, manganese chloride (MnCl_2_) and/or doxorubicin (also known as adriamycin) were diluted directly into the respective complete culture media at the indicated concentrations. All treatment solutions were prepared fresh from stock solutions prior to each experiment.

### 2.2 CCK-8 cell activity assay

To evaluate the effects of manganese chloride (MnCl_2_) on the viability of hepatocellular carcinoma cells and normal hepatocytes, Huh7 and MIHA cells were seeded in 96-well plates at a density of 3×10³ cells/well, while HepG2.2.15 cells were plated at 6×10³ cells/well. Following 24 h incubation under standard culture conditions, cells were exposed to complete medium containing MnCl_2_ in two-fold increasing concentrations (1, 2, 4, 8, 16, 32 μM). Differential treatment durations were implemented: 48 h for Huh7 and MIHA cells versus 72 h for HepG2.2.15 cells. Post-treatment, the medium was aspirated and replaced with fresh medium containing 10% CCK-8 reagent. After 2 h incubation at 37°C, absorbance was measured at 450 nm using a microplate reader.

To investigate the impact of MnCl_2_ on doxorubicin sensitivity, we seeded the cells at the same density as described above and established various experimental groups. Control groups, doxorubicin-treated groups, and groups cotreated with doxorubicin and MnCl_2_ at concentrations of 1 μM, 2 μM, 4 μM, 8 μM, 16 μM, or 32 μM were established. For the Huh7 cell line, doxorubicin was added to all groups except the control group to achieve a final concentration of 0.1 μg/ml. For the HepG2.2.15 cell line, doxorubicin was added to all groups except the control group to achieve a final concentration of 0.5 μg/ml. Cell viability was assessed in Huh7 cells after 48 h of treatment and in HepG2.2.15 cells after 72 h of treatment via the same methods as previously described.

To validate the time-dependent reduction in doxorubicin sensitivity induced by MnCl_2_, Huh7 and HepG2.2.15 cells were seeded into 96-well plates at densities of 1.2 × 10^4^ and 2.4 × 10^4^ cells per well, respectively. Following a 24-hour incubation, Huh7 cells in the co-treatment group were exposed to medium containing 0.5 μg/mL doxorubicin and 1 μM MnCl_2_, while the doxorubicin-only group received medium with 0.5 μg/mL doxorubicin. For HepG2.2.15 cells, the co-treatment group was treated with medium containing 1 μg/mL doxorubicin and 1 μM MnCl_2_, and the doxorubicin-only group received 1 μg/mL doxorubicin. Cell viability was subsequently assessed at designated time points using methods described previously.

### 2.3 Extended co-treatment and morphological analysis

Huh7 cells were seeded in 96-well plates and treated with a gradient of MnCl_2_ (1-32 μM) in combination with doxorubicin. After 72 hours, the culture medium in all groups was replaced with fresh medium containing the corresponding original concentrations of MnCl_2_ and doxorubicin. Following a total of 5 days of incubation, cell viability was quantitatively assessed using the CCK-8 assay according to the manufacturer’s instructions. Additionally, phase-contrast images were captured throughout the period to document morphological changes.

### 2.4 EdU assays of cell proliferation capacity

The Huh7 and HepG2.2.15 cell lines were divided into control, MnCl_2_, doxorubicin, and MnCl_2_/doxorubicin cotreatment groups. Huh7 cells were seeded at a density of 3000 cells per well in 96-well plates, and HepG2.2.15 cells were seeded at a density of 6000 cells per well in 96-well plates. In the MnCl_2_ treatment group, MnCl_2_ was added at a concentration of 1 μM. In the doxorubicin treatment group, doxorubicin was added to the Huh7 cell culture until the concentration of doxorubicin in the medium reached 0.1 μg/ml. In the cotreatment group, doxorubicin and MnCl_2_ were added until a doxorubicin concentration of 0.1 μg/ml and a MnCl_2_ concentration of 1 μM were reached. In HepG2.2.15 cells, the doxorubicin concentration was 0.5 μg/ml in the doxorubicin group and 1 μM in the MnCl_2_ group, and in the cotreatment group, the concentration was adjusted to 0.5 μg/ml doxorubicin and 1 μM MnCl_2_. The working solution was added to the well plates that had completed the intervention to bring the final concentration of the EdU working solution to 10 μM; the plates were then incubated for 2 h. The medium was then removed, 1 ml of fixation solution was added, and the cells were fixed for 15 min at room temperature. After the fixative was removed, wash solution was added, and the cells were washed three times for 5 min each time. After washing, the wash solution was removed, permeabilization solution was added, and the mixture was incubated for 10–15 minutes at room temperature. Two more washes were then performed for 4 minutes each. After the wash solution was removed, the reaction mixture was added, the mixture was shaken well and incubated for 30 minutes. At the end of incubation, the samples were washed again and then examined by fluorescence microscopy.

### 2.5 Detection of apoptosis by flow cytometry

To assess the impact of MnCl_2_ on the apoptosis of hepatocellular carcinoma cells using flow cytometry, Huh7 cells were seeded at a concentration of 2.4 × 10^5^ cells/well, and HepG2.2.15 cells were seeded at a concentration of 3.6 × 10^5^ cells/well in six-well plates. After 24 h, the original medium was aspirated from the wells. For the Huh7 cell line, the control group was supplemented with complete medium, the doxorubicin treatment group was treated with mixed medium containing 0.2 μg/ml doxorubicin, the MnCl_2_ treatment group was treated with mixed medium containing 1 μM MnCl_2_, and the cotreatment group was treated with mixed medium containing both 1 μM MnCl_2_ and 0.2 μg/ml doxorubicin. For the HepG2.2.15 cell line, the control group was supplemented with complete medium, the doxorubicin treatment group was treated with mixed medium containing 1 μg/ml doxorubicin, the MnCl_2_ treatment group was treated with mixed medium containing 1 μM MnCl_2_, and the cotreatment group was treated with mixed medium containing both 1 μM MnCl_2_ and 1 μg/ml doxorubicin. Apoptosis was assessed in Huh7 cells after 48 h of treatment and in HepG2.2.15 cells after 72 h of treatment. The detection method was performed according to the instructions provided with the apoptosis assay kit.

### 2.6 Eukaryotic transcriptome sequencing

We established two MnCl_2_ treatment groups (1 μM and 16 μM groups) and a control group. The cells treated with 1 μM MnCl_2_ were defined as MnCl_2_ treatment group 1, and those treated with 16 μM MnCl_2_ were defined as MnCl_2_ treatment group 2. Huh7 cells were seeded in 6-well plates at 2.4 × 10^5^ per well and cultured for 24 h, after which culture medium containing different concentrations of MnCl_2_ was added according to the group. After 48 h of intervention, the cells were harvested and total RNA was isolated using TRIzol (Invitrogen Life Technologies, USA) according to the manufacturer’s instructions. RNA was stored at -80°C after which all samples were transferred on dry ice to Nanjing Parnassus Biologicals for sequencing.

The mRNA with a polyA structure in the total RNA was enriched with oligo(dT) magnetic beads, the first strand of cDNA was synthesized with random 6-base primers and reverse transcriptase using the RNA as a template, and the second strand of cDNA was synthesized with first-strand cDNA as a template. After library construction, PCR amplification was used for library fragment enrichment, followed by library selection according to fragment size, with a library size of 450 bp. Next, the library quality was verified using an Agilent 2100 Bioanalyzer, and the total library concentration and effective library concentration were subsequently tested. The libraries containing different index sequences were mixed in proportion to the effective library concentration and the amount of data required for the library. The mixed libraries are uniformly diluted to 2 nM and formed into single-stranded libraries by alkali denaturation. After RNA extraction, purification, and library construction, these libraries are sequenced via next-generation sequencing (NGS) using the Illumina sequencing platform with paired-end (PE) sequencing.

### 2.7 Differential gene expression analysis and screening for MnCl_2_ functional marker genes

We used HTSeq statistics to compare the read count value of each gene to the raw expression of the gene. We used FPKM to normalize the expression (normalization) so that the gene expression levels were comparable across genes and samples. We then used DESeq for differential analysis of gene expression with the following conditions for screening the differentially expressed genes: expression difference multiplicity |log2FoldChange| > 1 and significance *P* value <0.05. We subsequently screened the MnCl_2_ functional marker genes that were significantly different in each group using the support vector machine (SVM) method and verified the discriminatory efficacy of these marker genes via multicategorical receiver operating characteristic (ROC) curves.

### 2.8 Validation of marker gene protein expression via ELISA

Using phosphate-buffered saline (PBS), we prepared suspensions of cells that had been treated with various concentrations of MnCl_2_. The protein was subsequently extracted via ultrasonic cracking, and the freeze–thaw process was repeated. Thereafter, the protein concentration was determined by the BCA method (in accordance with the manufacturer’s instructions). The experimental setup included blank control wells, standard curve wells, and sample wells. The protein suspension was diluted to a suitable detection range according to the instructions, and 50 μl of the sample was precisely added to the microplate. After incubation at 37°C for 30 minutes, the plate was washed with a wash solution. Then, 50 μl of the enzyme-labelled reagent was added, and the incubation and washing steps were repeated. Finally, chromogenic agents A and B were added sequentially, followed by 10 minutes of color development in the dark. The reaction was stopped by the addition of stop solution, and the optical density (OD) at 450 nm was measured within 15 minutes.

### 2.9 Functional enrichment analysis

GO enrichment analysis using topGO was performed by calculating the *P* value (*P* value < 0. 05 for significant enrichment) according to the hypergeometric distribution method to identify GO terms that were significantly enriched for differentially expressed genes compared with the whole-genomic background, thus identifying the main biological functions associated with the differentially expressed genes. Based on the GO and KEGG enrichment results, the degree of enrichment was measured by the Rich factor, FDR value, and the number of genes enriched with a given GO term. In addition, we used GSVA to explore the functions and pathways that were altered in different treatment groups. Furthermore, GSVA was employed to assess pathway alterations, from which core genes were identified as those with the highest absolute fold-change and a significant adjusted p-value (*p*.adj < 0.05) in significantly enriched pathways for biological interpretation.

### 2.10 Bioinformatics analysis of drug sensitivity and screening of doxorubicin sensitivity in different MnCl_2_ treatment groups

The doxorubicin sensitivity score and gene expression profile data were obtained via CellMiner: datasets for the NCI-60 cell line database, and the correlation between the doxorubicin sensitivity score and the expression of each gene was subsequently calculated to screen for doxorubicin sensitivity-related genes with the criteria of a *p* value<0.05 and an absolute correlation coefficient >0.4. The genes differentially expressed after treatment with MnCl_2_ were intersected with the genes related to doxorubicin sensitivity via a Venn diagram. The genes whose expression was downregulated and positively correlated with doxorubicin sensitivity under MnCl_2_ treatment and those whose expression was upregulated and negatively correlated with doxorubicin sensitivity under MnCl_2_ treatment were defined as MnCl_2_-induced doxorubicin resistance genes. We then investigated the half-maximal inhibitory concentration (IC50) of the drug using the Cancer Genome Project (CGP) database to predict the effects of different concentrations of MnCl_2_ on drug sensitivity via the pRRophetic R package.

### 2.11 Identification of MnCl_2_-treated hub genes and exploration of the role of hub genes in prognosis

We screened the PPI action pairs containing differential genes with a score >0.95 from the direct database according to the results of the differential gene expression analysis. The final interrelationships between all target genes were obtained (*PPI.network.txt). The relationship network was constructed using Cytoscape software to screen the top 10 pivotal genes according to the hub genes. The prognostic value of the hub genes was assessed with the Kaplan–Meier plotter (www.kmplot.com) online database, which contains gene expression data and survival information for patients with HCC. Patient samples were divided into two groups according to the optimal splitting point, and Kaplan–Meier survival plots were used to assess the prognostic role of the hub genes in hepatocellular carcinoma.

### 2.12 Western blot analysis

Huh7 cells were seeded into a 6-well plate at a density of 2.4×10^5^ cells per well and divided into the MnCl_2_ group, the doxorubicin group, the combination group, and the control group. Each group was treated with 1 μM MnCl_2_, 0.1 μg/ml doxorubicin, or a combination of both drugs. To investigate the concentration-dependent effects of MnCl_2_ on AKT phosphorylation in hepatocellular carcinoma cells, Huh7 cells were exposed to MnCl_2_ at graded concentrations spanning 1 μM to 256 μM, representing a 4-fold serial dilution series (1, 4, 16, 64, and 256 μM). After 48 h of treatment, protein was extracted.

HepG2.2.15 cells were seeded into a 6-well plate at a density of 3.6×10^5^ cells per well and divided into the MnCl_2_ group, the doxorubicin group, the combination group, and the control group. Each group was treated with 1 μM MnCl_2_, 0.5 μg/ml doxorubicin, or a combination of both drugs. Protein was extracted after 72 h of treatment.

The protein was extracted using RIPA cell lysis buffer, and the corresponding protein bands were cut according to the number of lanes and were labelled. The samples were then subjected to sodium dodecyl sulfate–polyacrylamide gel electrophoresis (SDS–PAGE), transferred onto a polyvinylidene difluoride (PVDF) membrane, and blocked with rapid blocking solution for 15 minutes. The membrane was washed and then incubated with primary antibodies (AKT [#4691, CST], P-AKT [#4060, CST], and GAPDH [60004-1-Ig, Proteintech]) overnight at 4°C, followed by incubation with secondary antibodies (HRP-conjugated goat anti-rabbit IgG [GB23303, Servicebio] and HRP-conjugated goat anti-mouse IgG [GB23302, Servicebio]) at room temperature for 1 h. After the membrane was washed, enhanced chemiluminescence (ECL) reagent was used for visualization.

### 2.13 Extraction of differentially expressed MnCl_2_ target genes in HCC and analysis of their interactions via the string and genemania online tools

Gene expression information for hepatocellular carcinoma (HCC) was obtained from the TCGA (The Cancer Genome Atlas) public database. We used the limma package within R software to screen for differentially expressed genes in HCC. The screening criteria were set as |logFC|>1 and a false discovery rate (FDR) less than 0.05. We subsequently identified the differentially expressed MnCl_2_ target genes in HCC by intersecting them with the MnCl_2_ target genes obtained from transcriptome sequencing. Furthermore, to investigate the interactions of these differentially expressed MnCl_2_ target genes, we submitted them to the STRING database (http://www.String-db.org/) and utilized its analytical capabilities to explore the potential impact of these genes on biological functions. The PPI network was established and visualized via Cytoscape software. We utilized the Hub plugin to identify the major role-playing differentially expressed MnCl_2_ target genes in hepatocellular carcinoma and further explored their potential functions through GeneMANIA (http://genemania.org/).

## 3 Results

### 3.1 The impact of varying MnCl_2_ concentrations on the activity of HCC cells

**Figure 1:**
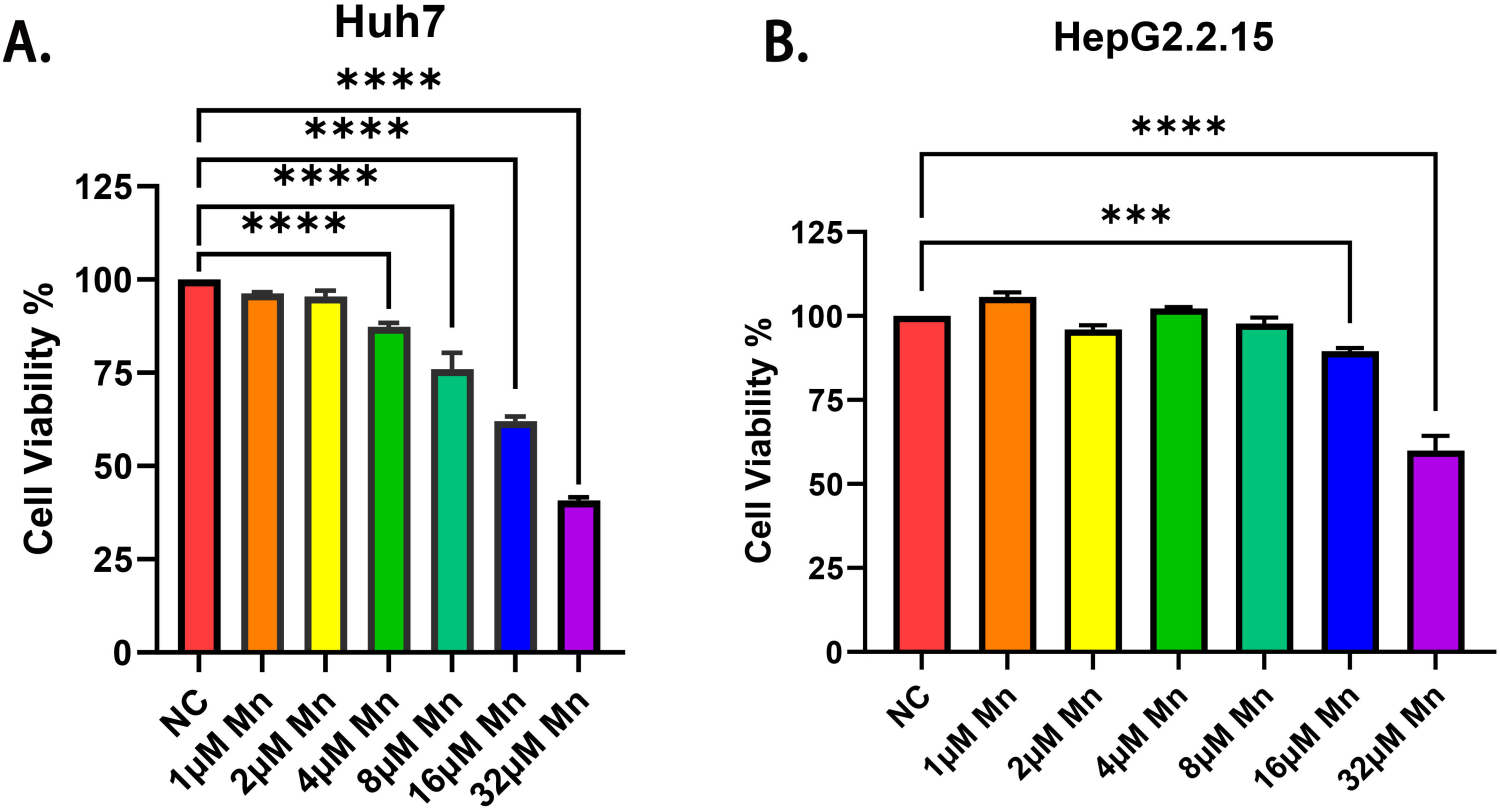
Effects of different MnCl_2_ concentrations on the activity of hepatocellular carcinoma cells. **(a)** Effects of different concentrations of MnCl_2_ on Huh7 cells. **(b)** Effects of different concentrations of MnCl_2_ on HepG2.2.15 cells. Statistical significance: ***=p < 0.001; **** =p < 0.0001.

In this study, we utilized a CCK-8 assay to investigate the impact of various MnCl_2_ concentrations on cell viability. We observed substantial inhibition of Huh7 cell viability upon exposure to MnCl_2_, with the inhibition commencing at a concentration of 4 μM (*p* < 0.01). Additionally, as the concentration of MnCl_2_ increased, a more pronounced inhibitory effect was observed (*p* < 0.0001). Compared with Huh7 cells, HepG2.2.15 cells exhibited greater resistance to Mn^2+^ induced cytotoxicity. A lower MnCl_2_ concentration (below 8 μM) had no significant effect on the viability of HepG2.2.15 liver cancer cells, whereas an attenuation of cell viability was observed when the concentration of MnCl_2_ was increased to 16 μM (*p* < 0.01) (**Figures 1A, B**). Furthermore, our study investigated the effects of various MnCl_2_ concentration gradients on both hepatocellular carcinoma cells (Huh7) and normal liver cells (MIHA). We observed that a MnCl_2_ concentration of 4μM was sufficient to significantly reduce the viability of Huh7 cells. In contrast, MIHA cells demonstrated a higher tolerance to MnCl_2_, with a significant inhibitory effect on their activity only observed at a much higher concentration of 32μM. These results highlight that hepatocellular carcinoma cells exhibit a greater sensitivity to Mn^2+^ (**Supplementary Figure 4**).

### 3.2 Low concentrations of MnCl_2_ decreases sensitivity of HCC to doxorubicin

**Figure 2:**
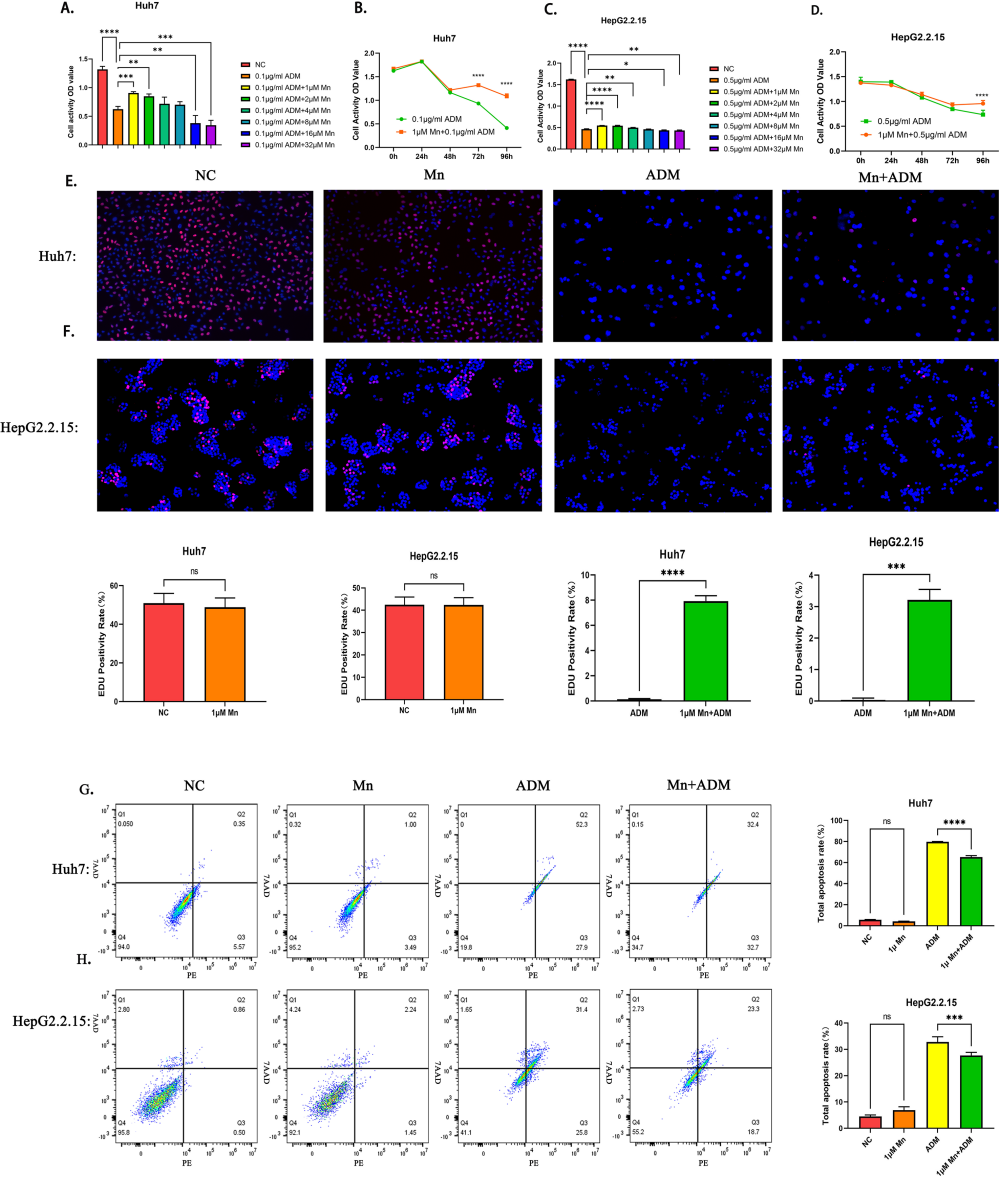
The role of low concentrations of MnCl_2_ in the induction of doxorubicin resistance in hepatocellular carcinoma cells. **(a)** Activity of Huh7 cells after cotreatment with doxorubicin and MnCl_2_ for 48 h. **(b)** Time-dependent induction of doxorubicin resistance in Huh7 cells by MnCl_2_. **(c)** Activity of HepG2.2.15 cells after 72 h of cotreatment with doxorubicin and MnCl_2_. **(d)** Time-dependent induction of doxorubicin resistance in HepG2.2.15 cells by MnCl_2_. **(e, f)** Effects of MnCl_2_ and doxorubicin treatment on the proliferative capacity of hepatocellular carcinoma cells. **(e)** Huh7, **(f)** HepG2.2.15. Proliferating cells are stained red with EdU, and nuclei are counterstained blue with DAPI. **(g, h)** Effects of MnCl_2_ and doxorubicin treatment on the apoptosis of hepatocellular carcinoma cells. **(g)** Huh7, **(h)** HepG2.2.15. Q1: The upper left quadrant represents cell debris without cell membranes or dead cells due to other causes; Q4: the lower left quadrant represents normal (living) cells; Q2: the upper right quadrant represents late apoptotic cells; and Q3: the lower right quadrant represents early apoptotic cells. Statistical significance: ns, p > 0.05; *, p < 0.05; **, p < 0.01;*** , p < 0.001; **** , p < 0.0001.

We observed that low concentrations of MnCl_2_ (1 μM and 2 μM) in combination with doxorubicin attenuate the sensitivity of HCC cells to doxorubicin (**Figures 2A, C**). Furthermore, we found that the reduction in doxorubicin sensitivity induced by MnCl_2_ is time-dependent. Diminished sensitivity to MnCl_2_-induced doxorubicin in Huh7 cells became evident after 72 hours of co-treatment (p < 0.0001), and a similar trend was observed in HepG2.2.15 cells, where reduced sensitivity was apparent only after 96 hours of MnCl_2_ treatment (p < 0.0001) (**Figures 2B, D**).

The resistance to doxorubicin induced by MnCl_2_ in Huh7 cells was noticeable only after 72 h of treatment (p < 0.0001), and a similar trend was observed in HepG2.2.15 cells, where resistance was evident after 96 h of MnCl_2_ treatment (p <0.0001) (**Figures 2B, D**).

Similarly, we verified the protective effect of MnCl_2_ by flow cytometry. The results revealed that low concentrations of MnCl_2_ did not promote the apoptosis of hepatocellular carcinoma cells but inhibited the apoptosis triggered by doxorubicin (**Figures 2G, H**) (Huh7: p <0.0001; HepG2.2.15: p < 0.001). Additionally, we performed an EdU assay to examine the impact of MnCl_2_ on the proliferation of hepatocellular carcinoma cells. Low concentrations of MnCl_2_ had no effect on cell proliferation. However, cotreatment with doxorubicin maintained the proliferative capacity of the Huh7 cell line to a certain extent (**Figures 2E, F**) (p < 0.01).

To further validate the long-term protective and cytotoxic effects of Mn^2+^, we conducted a 5-day co-treatment assay. Quantitative assessment confirmed that low-dose MnCl_2_ (1μM) sustained cell viability in the presence of doxorubicin, while high-dose MnCl_2_ (≥16μM) synergistically enhanced cytotoxicity (**Supplementary Figure 6**). Moreover, distinct morphological patterns suggested a potential shift in the mode of cell death (**Supplementary Figures 7**, **8**).

### 3.3 Differential transcriptomic and proteomic responses to MnCl_2_ exposure in HCC cells and identification of biomarkers

**Figure 3:**
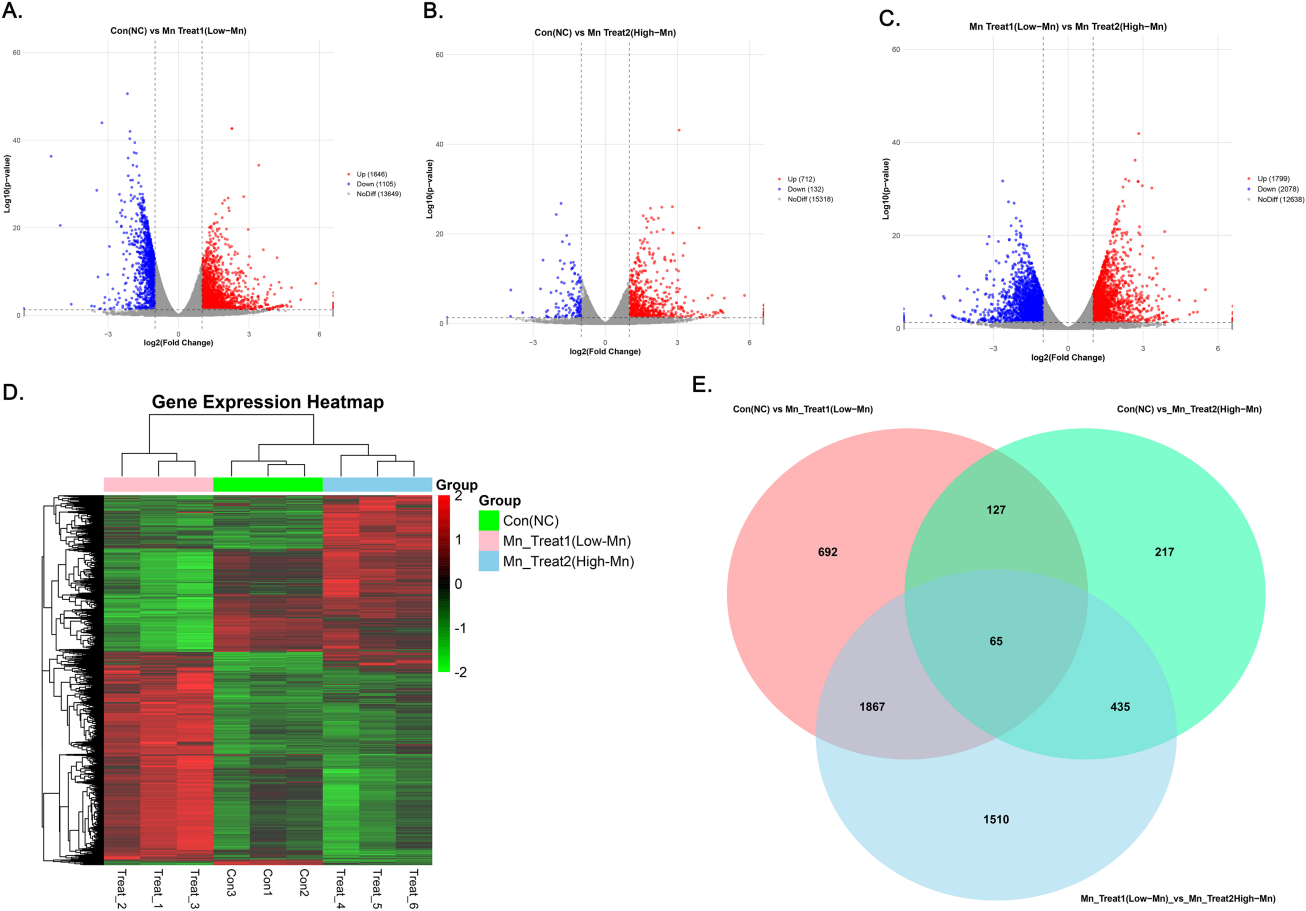
**(a)** Volcano plot of DEGs between the low-MnCl2 concentration treatment group and the control group. **(b)** Volcano plot of DEGs between the high-MnCl2 concentration treatment group and the control group. **(c)** Volcano plots of genes differentially expressed between the low- and high-MnCl2 treatment groups. Red represents genes with relatively high expression in that sample, and blue represents genes with relatively low expression in that sample. The horizontal coordinates represent the log2FoldChange multiplicity of the expression difference of the gene compared with the control, and the vertical coordinates represent the assumed value of -log10(p value). **(d)** Heatmap of gene expression under three different treatments. Red represents genes with relatively high expression in that sample, and green represents genes with relatively low expression in that sample. **(e)** Wayne plots of the three sets of DEGs. Con, Untreated control group; Mn Treat 1, experimental group treated with 1 μM MnCl2; Mn Treat 2, experimental group treated with 16 μM MnCl2.

We then investigated the impact of MnCl_2_ on hepatocellular carcinoma (HCC) cells. The heatmap (**Figure 3D**) revealed significant transcriptomic alterations in response to two distinct MnCl_2_ concentrations, which indicates that the treatments did not elicit identical effects on gene expression. The volcano plots (**Figures 3A-C**) revealed that, compared with the control group, the low-concentration MnCl_2_ treatment group (MnCl_2_ treatment group 1, 1 μM) presented upregulation of 1,646 genes and downregulation of 1,105 genes. Compared with the control group, the high-MnCl_2_ treatment group (MnCl_2_ treatment group 2, 16 μM) presented upregulation of 712 genes and downregulation of 132 genes. A comparison of the high- and low-MnCl_2_ treatment groups revealed upregulation of 1,799 genes and downregulation of 2,078 genes. Notably, 65 genes were differentially expressed across all three groups (**Figure 3E**).

**Figure 4:**
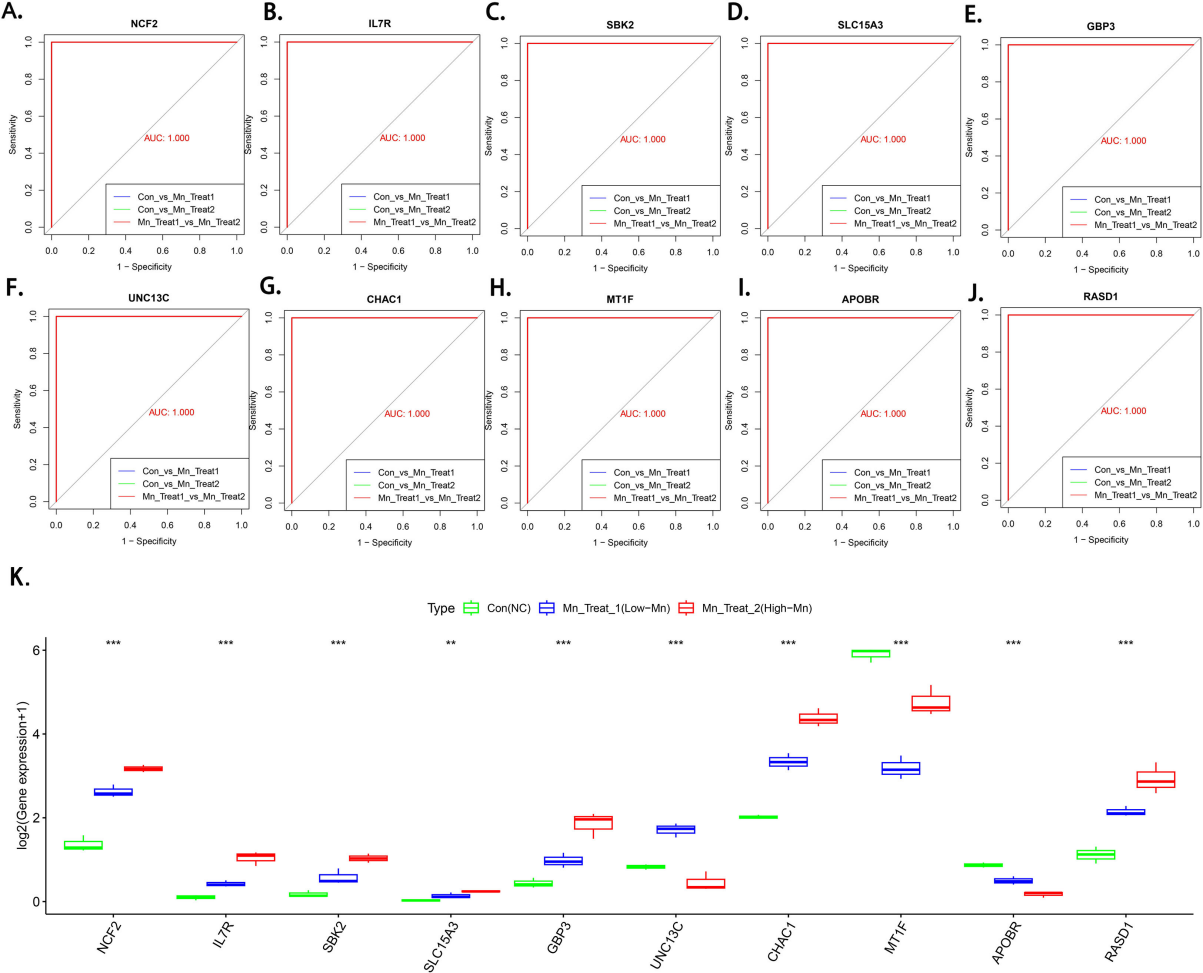
Screening of marker genes for different biological patterns of MnCl_2_-induced hepatocellular carcinoma cells. **(a-j)** Multiclassification ROC curves to validate marker gene classification efficacy. **(a)** NCF2, **(b)** IL7R, **(c)** SBK2, **(d)** SLC15A3, **(e)** GBP3, **(f)** UNC13C, **(g)** CHAC1, **(h)** MT1F, **(i)** APOBR, **(j)** RASD1, **(k)** Marker gene expression box plot.Con, Untreated control group; Mn Treat 1, experimental group treated with 1 μM MnCl_2_; Mn Treat 2, experimental group treated with 16 μM MnCl_2_. Statistical significance: ns, p > 0.05; *, p < 0.05; **, p < 0.01;***, p < 0.001; ****, p < 0.0001.

To understand the distinct initial cellular states induced by different concentrations of MnCl_2_ treatment, we employed a support vector machine (SVM) to screen for biomarkers capable of accurately identifying these three biological patterns. The identified biomarkers include NCF2, IL7R, SBK2, SLC15A3, GBP3, UNC13C, CHAC1, MT1F, APOBR, and RASD1. Multivariate ROC curve analysis demonstrated that these biomarkers achieved an Area Under the Curve (AUC) of 1 for distinguishing among the three treatment groups, indicating their exceptionally high accuracy in classifying Mn^2+^-induced initial cellular states (**Figures 4A-J**). Box plots further illustrate the expression patterns of these 10 biomarkers in their corresponding treatment groups (**Figure 4K**). Collectively, these biomarkers constitute a unique molecular signature of hepatocellular carcinoma cells under varying MnCl_2_ concentrations, laying the foundation for our subsequent in-depth analysis of the concentration-dependent protective and toxic mechanisms.

Additionally, we validated the differential expression of the molecular marker CHAC1 at the protein level in HCC cells under three distinct experimental conditions. Our findings indicate that the protein expression of CHAC1 was significantly elevated in HCC cells exposed to MnCl_2_ compared with control cells. Notably, the expression levels of CHAC1 increased with increasing concentrations of MnCl_2_. These results support the transcriptome sequencing data (**Supplementary Figure 3**).

### 3.4 GO functional enrichment analysis of HCC cells affected by different MnCl_2_ concentrations

**Figure 5:**
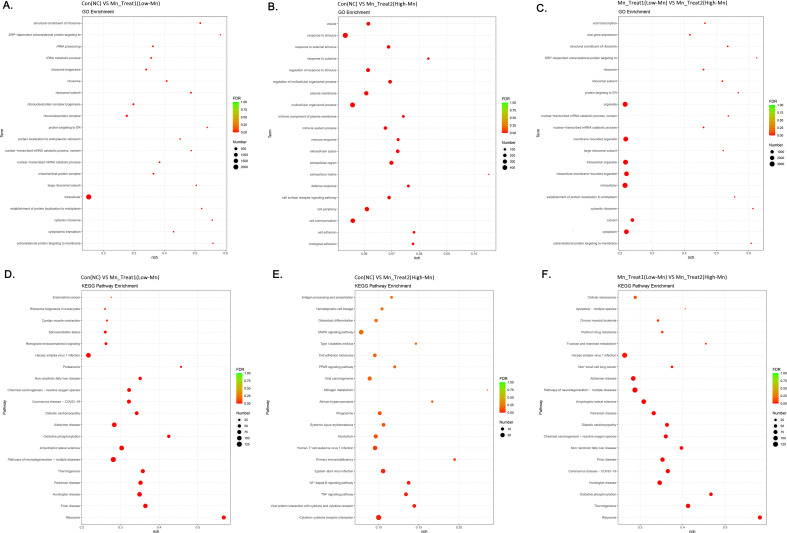
Functional enrichment analysis of genes differentially expressed after treatment with MnCl_2_. **(a-c)** GO enrichment analysis. **(a)** Con vs. Mn Treat 1. **(b)** Con vs. Mn Treat 2 **(c)** Mn Treat 1 vs. Mn Treat 2. **(d-f)** KEGG enrichment analysis. **(d)** Con vs. Mn Treat 1. **(e)** Con vs. Mn Treat 2 **(f)** Mn Treat 1 vs. Mn Treat 2. The size of the circle represents the number of genes enriched in the corresponding pathway or function. The gradation of color from green to red represents the change in the P value from large to small. Con, Untreated control group; Mn Treat 1, experimental group treated with 1 μM MnCl_2_; Mn Treat 2, experimental group treated with 16 μM MnCl_2_.

GO functional enrichment analysis revealed that the low-concentration MnCl_2_ treatment (Mn Treat 1, 1 μM) primarily affected processes related to transcription and translation, particularly ribosomal composition and function. These processes included ribosomes, ribosome biogenesis, rRNA metabolic processes, rRNA processing, mitochondrial protein complexes, nuclear transcription of mRNA breakdown metabolic processes, and cytoplasmic translation (**Figure 5A**). Conversely, the high-MnCl_2_ treatment group (Mn Treat 2, 16 μM) primarily influenced the cellular structure, such as cell vesicles, the matrix, and cell adhesion status, in addition to the cellular response to stimuli, cytokines and immune regulation (**Figure 5B**). The primary differences between the two MnCl_2_ concentration treatment groups were observed in the processes of cytoplasmic degradation, ribosome function, protein target interactions, and the metabolism of nuclear-transcribed mRNAs (**Figure 5C**).

### 3.5 KEGG pathway enrichment analysis of HCC cells affected by different MnCl_2_ concentrations

We also performed an enrichment analysis to explore the molecular mechanism by which MnCl_2_ alters cell function. The results of the KEGG enrichment analysis revealed that, compared with those of the control group, the results of the two MnCl_2_ treatment groups were different, and the samples in the low-MnCl_2_ treatment group were enriched mainly for ribosome, Huntington disease, Parkinson’s disease, thermogenesis, pathways of neurodegeneration, amyotrophic lateral sclerosis, oxidative phosphorylation, Alzheimer’s disease, chemical carcinogenesis, and reactive oxygen species (**Figure 5D**). Most of the enriched genes were related to various diseases and oxidative metabolism, and most were downregulated, which suggests that preventing the occurrence of these diseases at such concentrations may be possible (**Supplementary Table 1**).

The high-concentration MnCl_2_ treatment may cause changes in antigen processing and presentation, osteoclast differentiation, cell adhesion molecules, the PPAR signaling pathway, nitrogen metabolism, viral carcinogenesis, systemic lupus erythematosus, alcoholism, primary immunodeficiency, the NF−kappa B signaling pathway, the TNF signaling pathway, viral protein interactions with cytokines and cytokine receptors, and cytokine−cytokine receptor interactions. These findings suggest that the extensive activation of inflammation-related pathways at this concentration of MnCl_2_ may have affected the cellular immune response and invasion and migration ability under this treatment (**Figure 5E**).

Moreover, the enrichment analysis revealed substantial differences between the two MnCl_2_ treatment groups at different concentrations, mainly in terms of the KEGG pathways of cellular senescence, apoptosis, platinum drug resistance, fructose and mannose metabolism, Alzheimer’s disease, pathways of neurodegeneration, amyotrophic lateral sclerosis, Parkinson’s disease, diabetic cardiomyopathy, chemical carcinogenesis, non−alcoholic fatty liver disease, prion disease, and Huntington’s disease (**Figure 5F**).

The results of these enrichment analyses indicate that the differentially expressed genes under the two distinct MnCl_2_ concentrations are both enriched in neurological disorders (**Figure 5F**). However, the divergence lies in the fact that the majority of these genes, which are enriched in neurological disorders, are upregulated in cells treated with high concentrations of MnCl_2_, whereas they are downregulated in cells treated with low concentrations of MnCl_2_. Consequently, these two concentrations may elicit different outcomes in these diseases (**Supplementary Table 1**).

### 3.6 GSVA of the effects of different MnCl_2_ concentrations on the biological outcome of HCC cells

**Figure 6:**
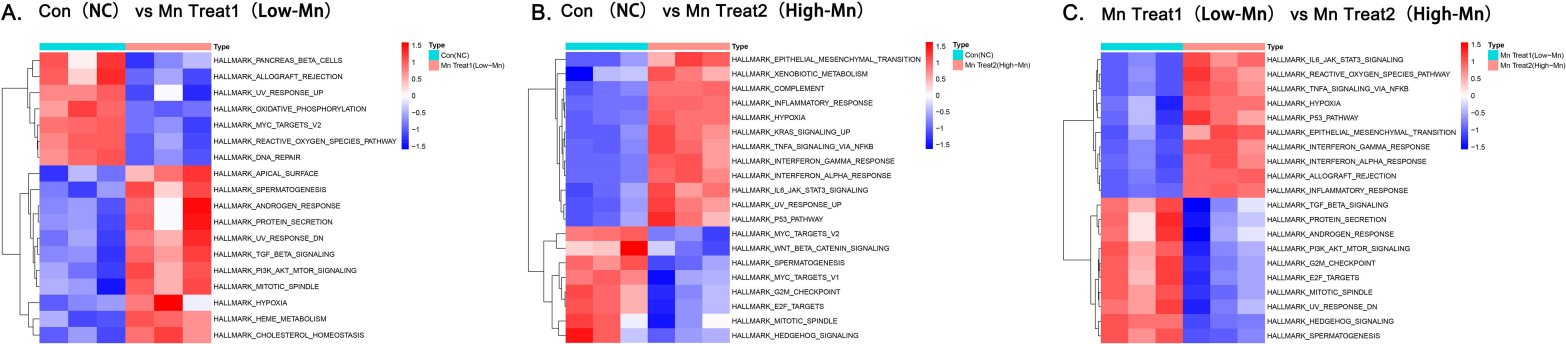
GSVA functional enrichment analysis of genes differentially expressed after treatment with MnCl_2_. **(a)** Con vs. Mn Treat 1. **(b)** Con vs. Mn Treat 2 **(c)** Mn Treat 1 vs. Mn Treat 2. The red grid represents relatively high expression within the group, the blue grid represents relatively low expression within the group, and the white grid represents no significant difference in expression. Con, Untreated control group; Mn Treat 1, experimental group treated with 1 μM MnCl_2_; Mn Treat 2, experimental group treated with 16 μM MnCl_2_.

GSVA revealed that in cells treated with low-dose MnCl_2_, gene sets associated with DNA damage repair, such as DNA repair, UV RESPONSE UP, and UV RESPONSE DN, were downregulated. In terms of tumor metabolism, oxidative phosphorylation, increased cholesterol homeostasis and heme metabolism were suppressed. Additionally, under this intervention, the reactive oxygen species (ROS) pathway and MYC pathway were inhibited, while the PI3K/AKT/mTOR signaling pathway, TGF-β signaling pathway, and the hypoxia pathway were activated. Notably, treatment with this dose of MnCl_2_ also induced an increase in cell mitosis (**Figure 6A**).

High concentrations of MnCl_2_ primarily targeted cell proliferation, immune activation, and pathway regulation. For example, the downregulation of MYC target pathways, E2F, the G2M checkpoint, and the mitotic spindle was observed. These biological signals are closely associated with cell cycle proliferation, which may be an important reason for the toxic effects of MnCl_2_ at this concentration on HCC cells. Furthermore, the increase in xenobiotic metabolism, inflammatory responses, interferon-γ responses, interferon-α responses, and IL6/JAK/STAT3 signaling suggests that MnCl_2_ treatment may be involved in modulating immune responses. Additionally, under this intervention, hypoxia, the KRAS signaling pathway, the P53 pathway, and the EMT pathway were upregulated, whereas the WNT/β-catenin and HEDGEHOG pathways were downregulated. These findings indicate that Mn^2+^ can broadly regulate the molecular functions of HCC cells (**Figure 6B**).

Compared with the low concentration of MnCl_2_, the high concentration of MnCl_2_ significantly activated immune and oxidative stress-related responses, such as interferon-γ (IFN-γ) responses, inflammatory reactions, and reactive oxygen species (ROS) pathways. In contrast, the low concentration of MnCl_2_ primarily activated cell cycle-related pathways, such as the G2M checkpoint and mitotic spindle (**Figure 6C**).

### 3.7 Key pathway analysis

Transcriptomic pathway analysis revealed that the cellular response to Mn^2+^ is highly concentration-dependent, activating sharply contrasting pro-survival and pro-death molecular programs. Under low MnCl_2_ (1µM), genes within the HALLMARK_PI3K_AKT_MTOR_SIGNALING pathway were significantly downregulated. Notably, these genes (e.g., PPP1CA, CALR, SFN, ARF1, PFN1) are known negative regulators of AKT, suggesting a derepression of this pro-survival axis. Conversely, high MnCl_2_ (16 µM) robustly activated stress and immune pathways. The HALLMARK_P53_PATHWAY showed upregulation of key effectors like DDIT4, SFN and SAT1, linking high Mn^2+^ to autophagy and ferroptosis. Simultaneously, the HALLMARK_INTERFERON_ALPHA_RESPONSE pathway was induced, marked by elevated interferon-stimulated genes (e.g., ISG15, IFITM3), indicating a potent innate immune response.

Thus, low Mn^2+^ may promote survival by potentiating AKT signaling, while high Mn^2+^ triggers integrated stress and immune responses. (A full gene list is in **Supplementary Tables 4**-**6**).

### 3.8 Low-dose Mn^2+^ induces lipid metabolic reprogramming independent of classical chemoresistance pathways

Given the established role of lipid metabolic reprogramming in chemoresistance, we analyzed our transcriptomic data for alterations in fatty acid and cholesterol synthesis pathways following low-dose MnCl_2_ treatment. Compared to the control, the low MnCl_2_ (1 µM) group exhibited a concerted upregulation of key genes involved in lipid metabolism (**Supplementary Table 7**). This included critical enzymes in *de novo* cholesterol biosynthesis, such as the rate-limiting HMGCR, as well as SQLE and HMGCS1. Concurrently, we observed significant induction of genes central to *de novo* fatty acid synthesis, including FASN, ACACA, and ACACB. Furthermore, genes implicated in lipid storage and droplet formation, such as LPIN1, LPIN2, and LPIN3, were also upregulated. This coordinated transcriptional program indicates that low-dose Mn^2+^ remodels hepatocellular carcinoma metabolism toward an anabolic state, a hallmark of drug-resistant cancer cells.

To further determine whether this metabolic shift aligns with classical multidrug resistance phenotypes, we performed Gene Set Variation Analysis (GSVA). While a significant enrichment of the KEGG_ABC_TRANSPORTERS pathway was observed (**Supplementary Figure 9**), the key multidrug resistance gene ABCB1 showed only marginal upregulation (logFC = 0.744). Similarly, although CPT1A—a rate-limiting enzyme in fatty acid β-oxidation—was significantly upregulated, GSVA revealed no concomitant activation of gene sets related to “fatty acid β-oxidation” or “glycerolipid metabolism” (**Supplementary Figure 10**). These findings collectively suggest that the lipid metabolic rewiring induced by low-dose Mn^2+^ represents a distinct adaptive mechanism, separable from canonical chemoresistance pathways.

### 3.9 Investigating the drug stratification effects of MnCl_2_ and identifying potential genes associated with decreased doxorubicin sensitivity

To further explore the specific molecular mechanism of MnCl_2_-induced doxorubicin resistance, we screened genes related to doxorubicin sensitivity using the CellMiner database. According to the above screening conditions, we ultimately screened 10 MnCl_2_-induced doxorubicin resistance genes, among which those with the highest correlation with doxorubicin sensitivity were ZNF662, SLC13A5, XDH, PTPN4, SMC1B, OR2A7, SLC35A3, ADCY5, UBB, and MYO6 (**Table 1**). Drug sensitivity analysis revealed that the sensitivity of MnCl_2_-treated hepatocellular carcinoma cells to drugs was dramatically altered (**Supplementary Table 2**). The sensitivity to doxorubicin was lower in the low- MnCl_2_ treatment group than in the other two groups, which is consistent with our previous findings.

**Table 1:** Screening for MnCl_2_-induced doxorubicin resistance genes. .

id	cor	pvalue
ZNF662	-0.569849177	2.01E-06
SLC13A5	-0.549740111	5.38E-06
XDH	-0.526115298	1.58E-05
PTPN4	-0.494476618	5.93E-05
SMC1B	-0.471982822	0.000140598
OR2A7	-0.455016769	0.000259561
SLC35A3	-0.429441086	0.00061704
ADCY5	-0.425739948	0.000695563
UBB	0.419908407	0.000837757
MYO6	-0.405841505	0.001294611

### 3.10 Identification of differentially expressed hub genes among various MnCl_2_ treatment groups and exploration of the significance of these genes in HCC prognosis

**Figure 7:**
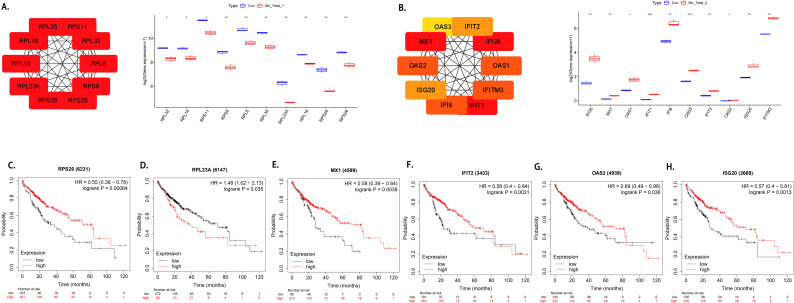
Hub genes in the group treated with two concentrations of MnCl_2_ and their effects on prognosis. **(a)** Hub genes associated with Mn Treat 1. **(b)** Hub genes associated with Mn Treat 2. **(c-g)** Mn^2+^ associated hub genes with prognostic impact. **(c)** RPS26, **(d)** RPL23A, **(e)** MX1, **(f)** IFIT2, **(g)** OAS2, **(h)** ISG20. Con, Untreated control group; Mn Treat 1, experimental group treated with 1 μM MnCl_2_; Mn Treat 2, experimental group treated with 16 μM MnCl_2_. Statistical significance: ns, p > 0.05; *, p < 0.05; **, p < 0.01;***, p < 0.001; ****, p < 0.0001.

We performed a protein-protein interaction analysis using the STRING database to reveal interactions between target genes. We subsequently screened 10 hub genes via the cytoHubba of Cytoscape, among which the hub genes in the low-MnCl_2_ treatment group were RPL32, RPL18, RPS11, RPS9, RPL8, RPL35, RPL23A, RPL19, RPS28, and RPS26. These 10 hub genes were expressed at higher levels in the control group (**Figure 7A**), whereas RPS26 and RPL23A are prognostic genes for hepatocellular carcinoma (**Figures 7C, D**). A survival analysis revealed that patients with hepatocellular carcinoma with high RPS26 expression had a better prognosis, whereas those with high RPL23A expression had a worse prognosis.

The hub genes in the high-MnCl_2_ treatment group were IFI35, MX1, OAS1, IFIT1, IFI6, OAS3, IFIT2, OAS2, ISG20, and IFITM3. These 10 hub genes were highly expressed in MnCl_2_ treatment group 2 (**Figure 7B**), where a survival analysis revealed that IFIT2, ISG20, MX1, and OAS2 could affect the prognosis of hepatocellular carcinoma patients (**Figures 7E-H**), and the high expression of these genes in patients was associated with a better prognosis.

### 3.11 A low concentration of MnCl_2_ induces AKT phosphorylation and modulates doxorubicin cytotoxicity in HCC cells

**Figure 8:**
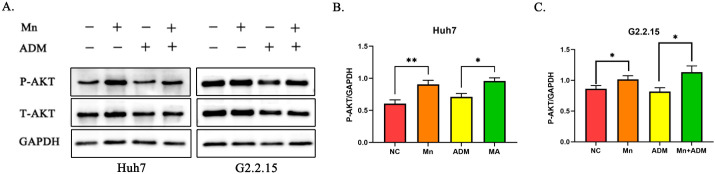
WB validation of the expression of phosphorylated AKT protein after MnCl_2_ and doxorubicin treatment. **(a)** WB protein bands showing AKT and AKT phosphorylation in Huh7 and HepG2.2.15 cells. **(b)** Histogram of the degree of relative AKT phosphorylation in Huh7 cells. **(c)** Histogram of the degree of relative AKT phosphorylation in HepG2.2.15 cells. The blot band plots shown for the Huh7 cell line are bands of the same molecular weight on different parts of the same gel. HepG2.2.15 shows the blot band plots derived from the same position on the same gel through the membrane regeneration solution. Statistical significance: ns, p > 0.05; *, p < 0.05; **, p < 0.01;***, p < 0.001; ****, p < 0.0001.

WB analysis demonstrated that a low concentration of MnCl_2_ treatment induces the phosphorylation and activation of AKT in Huh7 and HepG2.2.15 cells, a result that is consistent with previous gene set variation analysis (GSVA) findings. Moreover, the level of AKT protein phosphorylation in liver cancer cells cotreated with doxorubicin and MnCl_2_ was greater than that in those treated with doxorubicin alone. These findings suggest that the diminished cytotoxic effect of doxorubicin on liver cancer cells at this concentration of MnCl_2_ could be due to this underlying mechanism (**Figure 8**). Furthermore, we have also examined the impact of various MnCl_2_ concentrations on AKT phosphorylation. Our experimental findings demonstrate that at a MnCl_2_ concentration of 1μM, the ratio of P-AKT to GAPDH is markedly higher than that observed with higher MnCl_2_ doses of 16 and 250μM (**Supplementary Figure 5**).

### 3.12 Identification and functional analysis of MnCl_2_-responsive genes in HCC

By contrasting the differentially expressed genes identified in liver cancer versus normal liver tissue within the TCGA database against those targeted by MnCl_2_ from our sequencing data, we delineated a set of intersecting genes. Our analysis revealed 1,008 differentially expressed genes in hepatocellular carcinoma (HCC) in the low-MnCl_2_ treatment cohort and 201 differentially expressed genes in the high-MnCl_2_ treatment cohort (**Supplementary Table 3**).

Further exploration using the STRING database revealed the interaction networks of the differentially expressed MnCl_2_-targeted genes in HCC. Using Cytoscape, we identified 10 genes with significant functional implications. Within the low-MnCl_2_ treatment group, we identified potential interactions among RPL18, RPL8, RPL38, RPL10A, RPL37A, RPL7A, RPS11, RPL19, RPS14, and RPL13A, which may significantly influence endoplasmic reticulum function, ribosome biogenesis, and protein synthesis and modification pathways in HCC (**Supplementary Figure 1**).

Conversely, in the high-MnCl_2_ treatment group, we observed possible interactions among PSMB9, PSMB8, HLA-A, HLA-C, TAPBP, HLA-E, TAP1, IL1B, ALB, and SERPINE1, which are genes that likely modulate antigen processing and presentation, which are integral to the immune response in HCC (**Supplementary Figure 2**).

## 4 Discussion

This study unveils a previously underappreciated function of Mn^2+^ in cancer biology: the direct regulation of tumor cell chemosensitivity. Beyond its established potential as an immune adjuvant, we demonstrate that MnCl_2_, in a concentration-dependent manner, directly dictates the sensitivity of hepatocellular carcinoma cells to doxorubicin through intrinsic cellular pathways.

**Figure 9:**
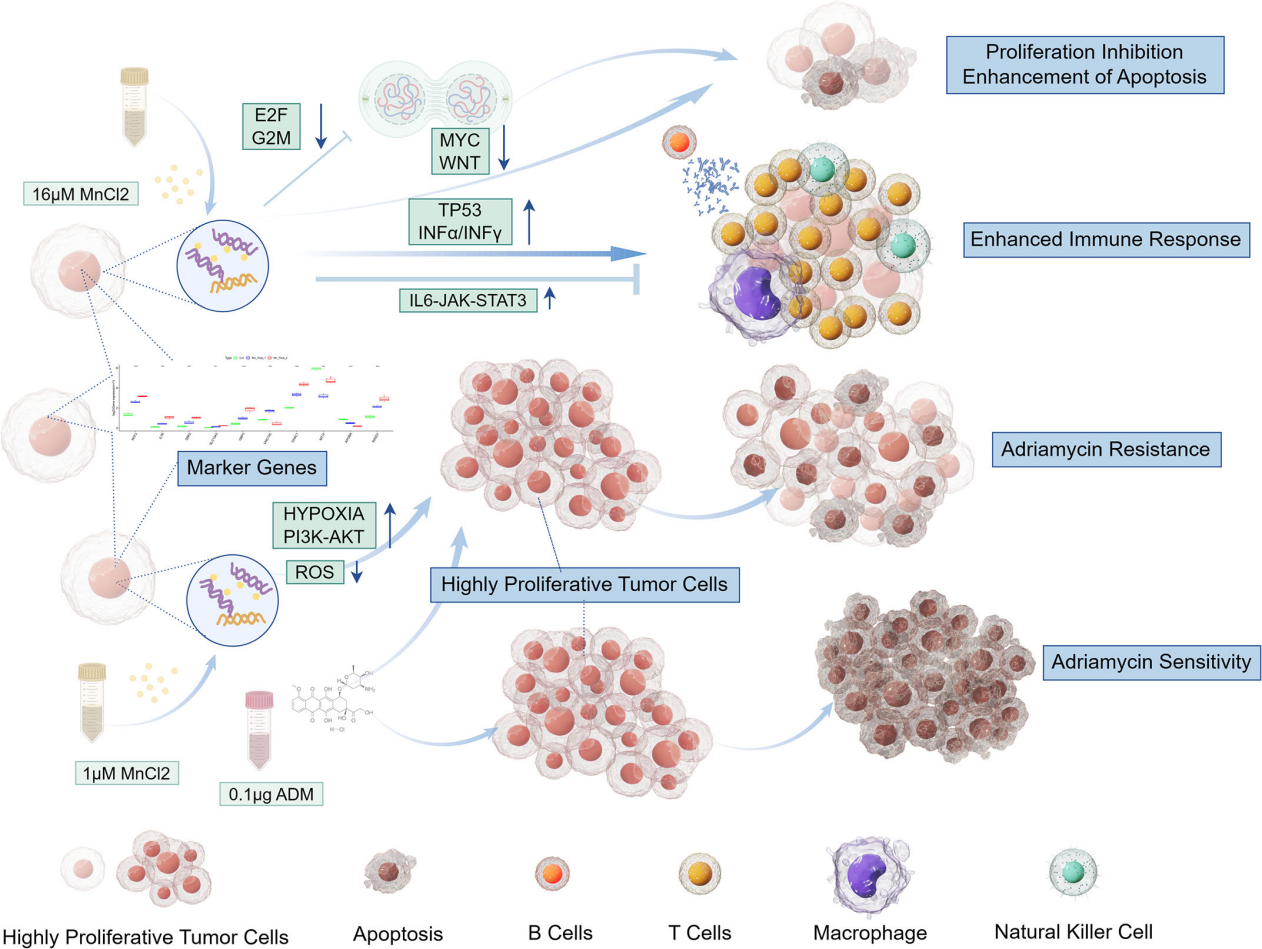
Effects of different concentrations of manganese chloride on hepatocellular carcinoma cells. At high concentrations, Mn^2+^ inhibits the proliferation of hepatocellular carcinoma cells by downregulating the E2F and G2/M cell cycle regulatory pathways, which in turn suppresses mitosis. Furthermore, Mn^2+^ impedes cell proliferation through downregulation of the MYC and WNT signaling pathways. In terms of the immune response, Mn^2+^ activates the TP53, interferon IFNγ and IFNα signaling pathways to increase immune cell activity. Concurrently, Mn^2+^ may also negatively modulate immune activation via the IL6-JAK-STAT3 signaling cascade. Conversely, at low concentrations, Mn^2+^ augments hepatocellular carcinoma cell resistance to the cytotoxic effects of doxorubicin. This is achieved by upregulation of the HIF and PI3K-AKT survival pathways and the concurrent downregulation of pathways associated with the production of ROS.

At low concentrations, Mn^2+^ promoted a chemoresistant phenotype by activating survival pathways such as PI3K-AKT and suppressing ROS production. In contrast, high concentrations induced a cytotoxic synergy with doxorubicin by arresting the cell cycle via E2F/G2M checkpoint downregulation and activating TP53. These direct effects on chemosensitivity represent a distinct layer of Mn^2+^ activity, complementing its concurrent—and more widely studied—modulation of immune pathways like interferon and IL6-JAK-STAT3 signaling. Our transcriptomic data, culminating in the identification of 10 molecular markers (**Figure 9**), solidify this dual and concentration-dependent role, positioning Mn^2+^ not merely as an immune stimulant but as a master regulator of the tumor cell’s response to chemical stress.

Although the MnCl_2_ concentrations used (1 and 16µM) exceed typical serum manganese levels—estimated at ∼70–270nM total Mn in healthy adults based on reported data (16)—they remain biologically relevant in the hepatic context. The liver is the primary site of manganese accumulation¹, and clinical studies consistently show that hepatocellular carcinoma (HCC) tissues are markedly depleted of manganese compared to adjacent non-tumor liver (5). Thus, 1µM may reflect the Mn-deficient tumor microenvironment, while 16µM approximates residual physiological manganese levels in liver tissue. Importantly, clinically feasible Mn²^+^ regimens have been shown to achieve micromolar-range systemic exposure and synergize with immune checkpoint blockade *in vivo (*12). Critically, even if systemic concentrations remain low, local Mn²^+^ enrichment within tumors could be attainable through emerging delivery strategies such as Mn²^+^-loaded nanocarriers or ionophore-assisted transport, which are designed to enhance intratumoral bioavailability.

The functional enrichment analysis of the low-MnCl_2_ treatment group revealed that Mn^2+^ is associated with neurological disorders, which is consistent with the findings of previous studies. Additionally, GSVA analysis indicated that low concentrations of Mn^2+^ may promote hypoxia-related pathways, increase the synthesis of cholesterol and hemoglobin, and downregulate the ROS pathway. Previous research has confirmed that the activation of hypoxia-related pathways and the downregulation of ROS pathways can induce resistance to doxorubicin (17, 18). Intriguingly, our GSVA analysis further revealed that low-dose Mn^2+^ treatment led to the downregulation of both MYC target pathways and reactive oxygen species (ROS) response pathways. This shift in transcriptional signature holds significant biological implications. As a potent driver of cellular processes, reduced MYC activity alleviates intrinsic proliferative pressure (19, 20), while diminished ROS signaling indicates a decline in intracellular oxidative stress. Collectively, these changes may enable tumor cells to transition from a state characterized by high proliferation and high stress toward a more stabilized, survival-oriented phenotype, thereby fostering a cellular milieu conducive to survival advantage under doxorubicin-induced cytotoxicity. The increased synthesis of cholesterol and hemoglobin may be involved in providing energy for tumors and promoting tumor progression (21, 22). This may serve as an explanation for the protective effect of MnCl_2_.

Our findings delineate a concentration-dependent duality for Mn^2+^ in hepatocellular carcinoma. At low doses, Mn^2+^ promotes a pro-survival phenotype, effectively augmenting HCC cell resistance to the cytotoxic effects of doxorubicin. Notably, the protective effect of low-dose Mn^2+^ in HepG2.2.15 cells was more pronounced in preserving proliferative capacity (EdU assay, **Figure 2F**) than in restoring overall metabolic activity (CCK-8 assay, **Figures 2C, D**). This distinction suggests that Mn^2+^ may preferentially protect the DNA replication machinery over general cellular metabolism under doxorubicin stress. The EdU assay, as a direct measure of DNA synthesis, specifically captures cells that successfully bypass S-phase arrest, while CCK-8 reflects broader metabolic states, including viable but non-proliferating cells. Thus, the robust EdU signal underscores that the core protective effect of low-dose Mn^2+^ is the maintenance of proliferative potential, a finding particularly relevant in the unique context of HBV-positive HepG2.2.15 cells.

Mechanistically, this protective effect is underpinned by the transcriptomic derepression of the PI3K-AKT pathway. The concerted downregulation of key negative regulators of AKT, such as PPP1CA and CALR, provides a molecular basis for the potentiation of this pro-survival axis, which is a well-established driver of chemoresistance (23, 24). Concurrently, the downregulation of SFN further contributes to a cellular environment conducive to proliferation. This orchestrated dampening of inhibitory signals likely converges to activate the AKT pathway—thereby promoting DNA repair and replication, enhancing cell survival, and reducing drug sensitivity, effects that may be amplified in HBV-related HepG2.2.15 cells due to virus-induced alterations in cellular stress responses (25).

In addition to PI3K-AKT pathway activation, we identified a concurrent metabolic reprogramming characterized by coordinated upregulation of genes involved in *de novo* cholesterol synthesis (HMGCR, SQLE), fatty acid synthesis (FASN, ACACA), and lipid storage (LPINs). This anabolic shift likely supports chemoresistance by enhancing membrane integrity and expanding energy reservoirs. However, GSVA-based analysis of classical chemoresistance pathways indicated that although the ABC transporter pathway was broadly altered, upregulation of the key multidrug resistance protein P-gp (ABCB1) remained limited. Furthermore, no systematic activation of canonical resistance-related processes such as fatty acid β-oxidation was detected, despite an isolated increase in CPT1A expression. Thus, the survival phenotype likely results from synergistic interactions between PI3K/AKT/mTOR-driven survival signaling and this early-stage, incompletely coordinated adaptation of specific lipid metabolic pathways. Future studies are needed to dissect the relative contributions and crosstalk among these distinct mechanisms. Furthermore, previous studies have shown that TGF activation plays a dual role in the development of HCC. Transforming growth factor-β can counteract proliferative stimuli by inducing cell apoptosis and senescence (26–28), but once tumor cells evade the growth inhibitory effects of TGF-β through genetic and epigenetic changes, tumor progression is promoted (29, 30).

Furthermore, we have identified genes induced by Mn^2+^ that are negatively correlated with doxorubicin sensitivity. Among them, UBB and XDH have been reported in the literature. In relevant studies, UBB was significantly downregulated in doxorubicin-resistant strains of breast cancer (31). Xanthine dehydrogenase (XDH) is an enzyme that can decrease the efficacy of chemotherapy drugs and plays different roles in aerobic and anaerobic environments. Under aerobic conditions, XDH activation leads to a higher rate of oxygen free radical formation than XO, which enhances the cytotoxicity of doxorubicin in aerobic tumor cells. However, under hypoxic conditions, it can promote the inactivation of doxorubicin through metabolic processes (32). GSVA of the low-dose MnCl_2_-treated group suggested enrichment of the HIF pathway. Hence, we speculate that Mn^2+^ may contribute to the reduction of HCC sensitivity to doxorubicin by enhancing the synergistic effects of HIF and XDH.

Enrichment analysis of the high-dose MnCl_2_-treated group revealed that Mn^2+^ may affect the adhesion of liver cancer cells, induce EMT, and promote the invasive migration of tumor cells. This finding is consistent with the discovery by Cardoso SC and Chebassier N regarding Mn^2+^-induced invasive migration (14, 33). Our study also revealed that higher concentrations of Mn^2+^ may downregulate the E2F, G2M, and WNT-related pathways. Previous studies have shown that downregulation of these pathways can inhibit HCC progression (34–36). Additionally, high-dose Mn^2+^ intervention can also downregulate the MYC pathway. Previous studies have indicated that MYC can promote the proliferation and migration of HCC cells as well as sorafenib resistance (37, 38). These findings provide a reasonable explanation for the toxicity and inhibitory effects of Mn^2+^ on HCC.

The toxic effects of Mn^2+^ on healthy organs can’t be ignored. Studies have shown that excessive Mn^2+^ may lead to neuroinflammation and neuronal damage, hepatotoxicity, lung damage, and increased permeability of glomerular endothelial cells (39–43). In our study, normal liver cells were found to tolerate higher MnCl_2_ concentrations. This suggests that Mn^2+^ can suppress tumor - cell killing at relatively safe doses. Clinical research has shown that using Mn^2+^ can enhance the effectiveness of immunotherapy (12). Also, certain designs might reduce Mn^2+^ toxicity to healthy tissues. For example, local drug delivery through transarterial chemoembolization (TACE) can achieve higher drug concentrations in the tumor area while minimizing effects on surrounding normal tissues (44). Encapsulating Mn^2+^ in nanoparticles is another feasible way to reduce toxicity (45).

Furthermore, our transcriptomic analysis delineates a complex immunomodulatory landscape triggered by high concentrations of MnCl_2_. This is most strikingly evidenced by the marked upregulation of classic interferon-stimulated genes (ISGs), including ISG15 and IFITM3, which drove the significant enrichment of the interferon-alpha and gamma response pathways in our GSVA. This finding aligns with the established role of Mn^2+^ as a cGAS-STING agonist and confirms its potent immune-activating properties (46, 47). Simultaneously, the significant activation of the TP53 pathway, underscored by the upregulation of effector genes like DDIT4 (48) and SAT1 (49), contributes to this integrated stress and immune response. However, the immune environment is not unidirectional. Our data also reveal a concurrent activation of the IL6/JAK/STAT3 and TNF pathways, which are widely recognized for their immunosuppressive functions in the tumor microenvironment (50–53). This apparent paradox may be explained by a built-in negative feedback mechanism; while manganese potently activates the cGAS-STING axis, this very activation can subsequently promote STAT3 activity to temper the initial immune activation, particularly of NK cells (54). Therefore, beyond its immune-enhancing effects, our findings suggest that under high MnCl_2_ treatment, hepatocellular carcinoma cells might engage immune escape mechanisms via this IL6/JAK/STAT3-mediated negative feedback loop. These insights are crucial for the potential application of Mn^2+^ in immunotherapy, as they highlight the necessity to co-target these compensatory immunosuppressive pathways to achieve a durable anti-tumor immune response.

The enriched pathways according to the transcriptomic analysis have also been confirmed in other studies. Mn^2+^ has been shown to activate the JAK2-STAT3, TNF, NF-KB, PI3K-AKT, and p53 pathways in various models (55–58). Research has indicated that a dose of 10 mg/kg Mn^2+^ can affect prepubertal kisspeptin and LHRH through mTOR pathway activation (59). However, exposure to high doses of Mn^2+^ can inhibit mTOR expression, which induces hepatic oxidative stress and autophagy in chickens. This effect may be dose dependent. Additionally, studies have reported that Mn^2+^ deficiency may be associated with the downregulation of TGF, whereas excessive Mn^2+^ intake may be related to MYC downregulation (60, 61). These findings are consistent with our transcriptomic pathway analysis.

Finally, we identified the hub genes that play major roles in affecting the biological behavior of liver cancer cells at different concentrations of MnCl_2_. Among the hub genes in the low-concentration MnCl_2_ group, RPL23A and RPS26 were found to impact the prognosis of patients with HCC. Previous studies have suggested that RPL23A may be an inducer of HCC, while inhibiting RPS26 may promote proliferation (62–64). Similarly, in the high-concentration MnCl_2_ group, OAS2, IFIT2, ISG20, and MX1 were found to impact the prognosis of HCC patients. Previous research has shown that IFIT2 can promote cell death, whereas OAS2 is associated with a favorable prognosis in patients with breast cancer (65, 66), which aligns with our results. However, studies have also indicated that MX1 and ISG20 are associated with a poorer prognosis (67–69). Therefore, further research is needed to understand the role of these gene expression changes in HCC. We have identified biomarkers that emerge under different concentration patterns of MnCl_2_, which may aid in distinguishing between the cytotoxicity induced by Mn^2+^ at high concentrations and the reduced sensitivity to doxorubicin at low concentrations.

These findings may provide a molecular basis for future research and offer new insights into the treatment and molecular mechanisms of MnCl_2_ in HCC. We acknowledge that the current study, being conducted *in vitro*, has inherent limitations in recapitulating the complexity of an *in vivo* tumor microenvironment. The intriguing dual role of Mn^2+^ concentration uncovered here provides a compelling rationale for future investigations in animal models. As rightly suggested, establishing xenograft models to delineate the effects of Mn^2+^/doxorubicin combinations on tumor growth and immune infiltration will be an essential next step in validating and translating these mechanistic insights.

## Data Availability

The datasets presented in this study can be found in online repositories. The names of the repository/repositories and accession number(s) can be found in the article/**Supplementary Material**.
