# Supplementary material for: Manganese modulates hepatocellular carcinoma cytotoxicity and doxorubicin sensitivity in a dose dependent manner
Source: Front Oncol. 2026 Feb 13;16:1715702. doi: 10.3389/fonc.2026.1715702 (PMC12946836; doi:10.3389/fonc.2026.1715702)
Supplement: Supplementary file 1 [file DataSheet1.pdf]

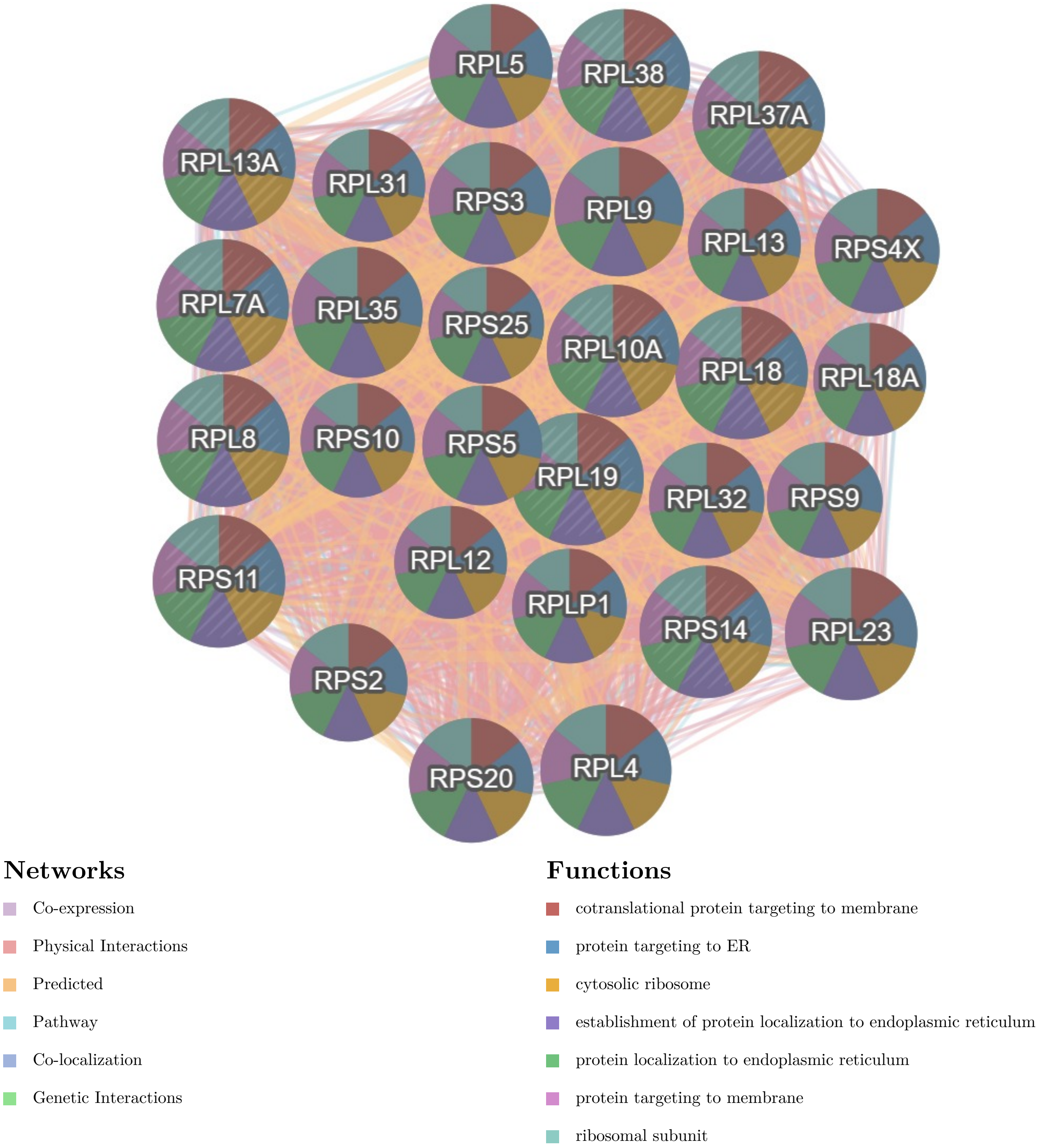

Supplementary Figure 1：The protein-protein interaction network of hub genes at the intersection of low concentration manganese target genes and diff erentially exp ressed genes in HCC and normal liver tissues.The different colors of the network edges indicate the bioinformatics methods applied: co-expression, site prediction, pat hways, physical interactions and co-localization. The different colors of the network nodes indicate the biological functions of the enriched genes.
