# Supplementary material for: Manganese modulates hepatocellular carcinoma cytotoxicity and doxorubicin sensitivity in a dose dependent manner
Source: Front Oncol. 2026 Feb 13;16:1715702. doi: 10.3389/fonc.2026.1715702 (PMC12946836; doi:10.3389/fonc.2026.1715702)
Supplement: Supplementary file 3 [file DataSheet3.pdf]

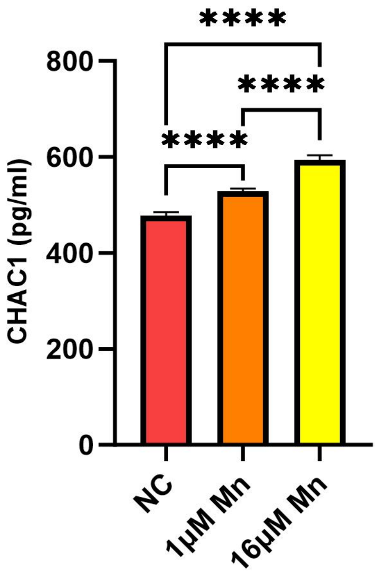

Supplementary Figure 3： Validation of CHAC1 Protein Expression via ELISA Assay.Red represents the expression level of CHAC1 in untreated Huh7 cells, orange represents the expression level of CHAC1 in Huh7 cells treated with 1μM manganese chloride, and yellow represents the expression level of CHAC1 in Huh7 cells treated with 16 μM manganese chloride.
