# Supplementary material for: Manganese modulates hepatocellular carcinoma cytotoxicity and doxorubicin sensitivity in a dose dependent manner
Source: Front Oncol. 2026 Feb 13;16:1715702. doi: 10.3389/fonc.2026.1715702 (PMC12946836; doi:10.3389/fonc.2026.1715702)
Supplement: Supplementary file 6 [file Presentation1.pdf]

The datasets generated and analyzed during the present study are available from the corresponding author on reasonable request. Eukaryotic transcriptome sequencing data have been uploaded to the GEO database. The link is https://www.ncbi.nlm.nih.gov/bioproject/PRJNA943337.
