# Supplementary material for: Manganese modulates hepatocellular carcinoma cytotoxicity and doxorubicin sensitivity in a dose dependent manner
Source: Front Oncol. 2026 Feb 13;16:1715702. doi: 10.3389/fonc.2026.1715702 (PMC12946836; doi:10.3389/fonc.2026.1715702)
Supplement: Supplementary file 9 [file Table3.docx]

**Supplementary Tables 4**

**The top genes with biological significance in the PI3K_AKT_MTOR pathway in the "NC group" and the "Low Mn group" GSVA analysis**

| **Gene** | **Pathway** | **Direction** | **logFC** | **adj.P.Val** | **Known Impact on Pathway** | **Brief Description of Biological Function** |
| --- | --- | --- | --- | --- | --- | --- |
| PPP1CA | HALLMARK_PI3K_AKT_MTOR_SIGNALING | Down | -199.53 | 0.0056 | Induces AKT dephosphorylation ^[1, 2]^ | 1.Promotes tumorigenesis^[3]^ 2.Involved in immune and inflammatory regulation ^[4]^ |
| CALR | HALLMARK_PI3K_AKT_MTOR_SIGNALING | Down | -152.51 | 0.0054 | The impact of CALR on the AKT pathway is not uniform . CALR mutations can directly activate serine/threonine kinase (AKT)^[5]^. In colorectal cancer ^[6]^and gastric cancer ^[7]^, CALR can suppress the AKT pathway and its phosphorylation. | Regulates endoplasmic reticulum homeostasis ^[8]^;CALR exhibits dual roles in immunity ^[9, 10]^ |
| PFN1 | HALLMARK_PI3K_AKT_MTOR_SIGNALING | Down | -147.63 | 0.0335 | Associated with inhibitory effect and downregulation ^[11, 12]^ | Recognizes RNA modifications ^[13]^; Negatively regulates CTL-mediated cytotoxicity ^[14]^ |
| ARF1 | HALLMARK_PI3K_AKT_MTOR_SIGNALING | Down | -80.3931128 | 0.0211 | Associated with inhibitory effect and negative regulation^[15]^ | Plays a dual regulatory role in cancer; ^[16, 17]^ |
| SFN | HALLMARK_PI3K_AKT_MTOR_SIGNALING | Down | -65.73851327 | 0.0102 | Exhibits bidirectional and context-dependent effects. Promotes activity in hepatocellular carcinoma cells ^[18]^. In lymphoma, SFN inhibits AKT substrate pS552 β-catenin phosphorylation, leading to its reduction ^[19]^ | Possesses antioxidant function ^[20]^; Exhibits anti-inflammatory function ^[21]^; Mediates tumor progression^[18]^ |

**References**

[1] Huang B, Ren J, Ma Q, et al. A novel peptide PDHK1-241aa encoded by circPDHK1 promotes ccRCC progression via interacting with PPP1CA to inhibit AKT dephosphorylation and activate the AKT-mTOR signaling pathway. Mol Cancer. 2024. 23(1): 34.

[2] Dedigama-Arachchige PM, Acharige N, Zhang X, Bremer HJ, Yi Z, Pflum M. Identification of PP1c-PPP1R12A Substrates Using Kinase-Catalyzed Biotinylation to Identify Phosphatase Substrates. ACS Omega. 2023. 8(39): 35628-35637.

[3] Liu X, Xu G, Luo W, Wang K, Wang F. PPP1CA promotes hepatocellular carcinoma progression in a YAP1-dependent way. Cell Signal. 2025. 134: 111938.

[4] Jiao X, Jiao Y, Cui J, et al. S100A4 targets PPP1CA/IL-17 to inhibit the senescence of sheep endometrial epithelial cells. Front Vet Sci. 2024. 11: 1466482.

[5] Wang C, Hu X, Wan Y, et al. The Synergistic Inhibitory Effect of Combining MK-2206 and AZD 6244 in MARIMO Cells Harboring a Calreticulin Gene Mutation. Chemotherapy. 2021. 66(5-6): 169-178.

[6] Jeong KY, Park M, Sim JJ, Kim HM. Combination Antitumor Effect of Sorafenib via Calcium-Dependent Deactivation of Focal Adhesion Kinase Targeting Colorectal Cancer Cells. Molecules. 2020. 25(22).

[7] Li H, Yang Z, Huang J, et al. CALCR interaction with ANTXR1 drives gastric tumor growth and metastasis via AKT signaling pathway. Sci Rep. 2025. 15(1): 11826.

[8] Fucikova J, Spisek R, Kroemer G, Galluzzi L. Calreticulin and cancer. Cell Res. 2021. 31(1): 5-16.

[9] Liu P, Zhao L, Loos F, et al. Immunosuppression by Mutated Calreticulin Released from Malignant Cells. Mol Cell. 2020. 77(4): 748-760.e9.

[10] Kepp O, Liu P, Zhao L, Plo I, Kroemer G. Surface-exposed and soluble calreticulin: conflicting biomarkers for cancer prognosis. Oncoimmunology. 2020. 9(1): 1792037.

[11] Bai N, Ma Y, Zhao J, Li B. Knockdown of lncRNA HCP5 Suppresses the Progression of Colorectal Cancer by miR-299-3p/PFN1/AKT Axis. Cancer Manag Res. 2020. 12: 4747-4758.

[12] Zhang S, Guo X, Liu X, Zhong Z, Yang S, Wang H. Adaptor SH3BGRL promotes breast cancer metastasis through PFN1 degradation by translational STUB1 upregulation. Oncogene. 2021. 40(38): 5677-5690.

[13] Wei S, Dai X, Yuan J, et al. Quantitative Proteomics Identifies Profilin-1 as a Pseudouridine-Binding Protein. J Am Chem Soc. 2025. 147(2): 1458-1462.

[14] Schoppmeyer R, Zhao R, Cheng H, et al. Human profilin 1 is a negative regulator of CTL mediated cell-killing and migration. Eur J Immunol. 2017. 47(9): 1562-1572.

[15] Luchsinger C, Aguilar M, Burgos PV, Ehrenfeld P, Mardones GA. Functional disruption of the Golgi apparatus protein ARF1 sensitizes MDA-MB-231 breast cancer cells to the antitumor drugs Actinomycin D and Vinblastine through ERK and AKT signaling. PLoS One. 2018. 13(4): e0195401.

[16] Ko A, Han SY, Song J. Regulatory Network of ARF in Cancer Development. Mol Cells. 2018. 41(5): 381-389.

[17] Hu HF, Gao GB, He X, et al. Targeting ARF1-IQGAP1 interaction to suppress colorectal cancer metastasis and vemurafenib resistance. J Adv Res. 2023. 51: 135-147.

[18] Li R, Yan X, Zhong W, et al. Stratifin promotes the malignant progression of HCC via binding and hyperactivating AKT signaling. Cancer Lett. 2024. 592: 216761.

[19] Zhang Y, Gilmour A, Ahn YH, de la Vega L, Dinkova-Kostova AT. The isothiocyanate sulforaphane inhibits mTOR in an NRF2-independent manner. Phytomedicine. 2021. 86: 153062.

[20] Lv Y, Jiang H, Li S, et al. Sulforaphane prevents chromium-induced lung injury in rats via activation of the Akt/GSK-3β/Fyn pathway. Environ Pollut. 2020. 259: 113812.

[21] Wu Y, Gao M, Wu J, et al. Sulforaphane triggers a functional elongation of microglial process via the Akt signal. J Nutr Biochem. 2019. 67: 51-62.
