# Supplementary material for: Manganese modulates hepatocellular carcinoma cytotoxicity and doxorubicin sensitivity in a dose dependent manner
Source: Front Oncol. 2026 Feb 13;16:1715702. doi: 10.3389/fonc.2026.1715702 (PMC12946836; doi:10.3389/fonc.2026.1715702)
Supplement: Supplementary file 10 [file Table4.docx]

**Supplementary Tables 5**

**The top genes with biological significance in the P53 pathway in the "NC group" and the "High Mn group" GSVA analysis**

| **Gene** | **Pathway** | **Direction** | **logFC** | **adj.P.Val** | **Known Impact on Pathway** | **Brief Description of Biological Function** |
| --- | --- | --- | --- | --- | --- | --- |
| S100A4 | HALLMARK_P53_PATHWAY | Up | 588.87 | 0.0043 | Primarily downregulates p53 activity ^[1]^ | Promotes tumor progression and metastasis ^[2]^; induces pro-inflammatory responses ^[3]^ |
| S100A10 | HALLMARK_P53_PATHWAY | Up | 187.90 | 0.0038 | indirect inhibition^[4]^ | Promotes apoptosis and autophagy ^[5]^; promotes inflammatory signal transduction ^[6]^ |
| DDIT4 | HALLMARK_P53_PATHWAY | Up | 54.89 | 0.0116 | Acts as a downstream factor of p53 and activates it ^[7]^ | Promotes ferroptosis ^[8]^ |
| PCNA | HALLMARK_P53_PATHWAY | Down | -41.06 | 0.0256 | Inhibits p53 pathway ^[9]^ | Regulates DNA replication, DNA repair ^[10]^ |
| SFN | HALLMARK_P53_PATHWAY | Up | 38.23 | 0.0385 | SFN can activate p53 signaling pathway ^[11]^ | Promotes cell apoptosis ^[12]^; induces cell cycle arrest^[13]^ |
| SAT1 | HALLMARK_P53_PATHWAY | Up | 27.39 | 0.0011 | Primarily activates p53 pathway^[14]^ | Pro-apoptotic and Anti-tumor effects^[15]^ |

References

[1] Xia H, Gilbertsen A, Herrera J, et al. Calcium-binding protein S100A4 confers mesenchymal progenitor cell fibrogenicity in idiopathic pulmonary fibrosis. J Clin Invest. 2017. 127(7): 2586-2597.

[2] Fei F, Qu J, Li C, Wang X, Li Y, Zhang S. Role of metastasis-induced protein S100A4 in human non-tumor pathophysiologies. Cell Biosci. 2017. 7: 64.

[3] D'Ambrosi N, Milani M, Apolloni S. S100A4 in the Physiology and Pathology of the Central and Peripheral Nervous System. Cells. 2021. 10(4).

[4] Harada Y, Ikeda S, Kawabe Y, et al. S100A4 contributes to colorectal carcinoma aggressive behavior and to chemoradiotherapy resistance in locally advanced rectal carcinoma. Sci Rep. 2024. 14(1): 31338.

[5] Chen YD, Fang YT, Chang CP, et al. S100A10 Regulates ULK1 Localization to ER-Mitochondria Contact Sites in IFN-γ-Triggered Autophagy. J Mol Biol. 2017. 429(1): 142-157.

[6] Bagheri-Hosseinabadi Z, Abbasi M, Kahnooji M, Ghorbani Z, Abbasifard M. The prognostic value of S100A calcium binding protein family members in predicting severe forms of COVID-19. Inflamm Res. 2022. 71(3): 369-376.

[7] Coronel L, Häckes D, Schwab K, Riege K, Hoffmann S, Fischer M. p53-mediated AKT and mTOR inhibition requires RFX7 and DDIT4 and depends on nutrient abundance. Oncogene. 2022. 41(7): 1063-1069.

[8] Chen Y, Feng X, Li Z, et al. Targeting ATF4-DDIT4/TXNIP induced mitochondrial dysfunction and ferroptosis: ISRIB as novel therapy for septic cardiomyopathy. J Transl Med. 2025. 23(1): 938.

[9] Reddy S, Doshi S, Pathengay A, Panchal B. Ocular decompression retinopathy following intracameral bevacizumab injection in a case of proliferative diabetic retinopathy with neovascular glaucoma. Indian J Ophthalmol. 2020. 68(6): 1206-1209.

[10] Wang T, Wang Z. Targeting the "Undruggable": Small-Molecule Inhibitors of Proliferating Cell Nuclear Antigen (PCNA) in the Spotlight in Cancer Therapy. J Med Chem. 2025. 68(3): 2058-2088.

[11] Beekman AM, Cominetti M, Walpole SJ, et al. Identification of selective protein-protein interaction inhibitors using efficient in silico peptide-directed ligand design. Chem Sci. 2019. 10(16): 4502-4508.

[12] Wang Y, Wu H, Dong N, et al. Sulforaphane induces S-phase arrest and apoptosis via p53-dependent manner in gastric cancer cells. Sci Rep. 2021. 11(1): 2504.

[13] Ren Z, Feng J, Yang M, Feng D, Wang X, Liu W. Mechanism of sulforaphane in treatment of pancreatic cancer cell based on network pharmacology and in vitro experiments. Bioorg Chem. 2025. 163: 108754.

[14] Liang T, Xie J, Zhao J, et al. Novel lnc-HZ03 and miR-hz03 promote BPDE-induced human trophoblastic cell apoptosis and induce miscarriage by upregulating p53/SAT1 pathway. Cell Biol Toxicol. 2021. 37(6): 951-970.

[15] Mou Y, Zhang L, Liu Z, Song X. Abundant expression of ferroptosis-related SAT1 is related to unfavorable outcome and immune cell infiltration in low-grade glioma. BMC Cancer. 2022. 22(1): 215.
