# Supplementary material for: Manganese modulates hepatocellular carcinoma cytotoxicity and doxorubicin sensitivity in a dose dependent manner
Source: Front Oncol. 2026 Feb 13;16:1715702. doi: 10.3389/fonc.2026.1715702 (PMC12946836; doi:10.3389/fonc.2026.1715702)
Supplement: Supplementary file 11 [file Table5.docx]

**Supplementary Tables 6**

**The top genes with biological significance in the IFNα pathway in the "NC group" and the "High Mn group" GSVA analysis**

| **Gene** | **Pathway** | **Direction** | **logFC** | **adj.P.Val** | **Known Impact on Pathway**  **(Activation/Inhibition)** | **Brief Description of Biological Function** |
| --- | --- | --- | --- | --- | --- | --- |
| ISG15 | HALLMARK_INTERFERON_ALPHA_RESPONSE | Up | 360.84 | 0.0204 | The core role of ISG15 in the IFN-α pathway is dynamic homeostasis: it indirectly inhibits excessive IFN-α signaling via USP18^[1]^, while secreted ISG15 enhances other immune effects (e.g., IFN-γ) ^[2]^ | 1. Antiviral and pro-inflammatory balance ^[3]^;  2. Highly expressed in cancers (e.g., pancreatic cancer), potentially involved in carcinogenesis or immune evasion ^[4]^ |
| PSME1 | HALLMARK_INTERFERON_ALPHA_RESPONSE | Up | 100.80 | 0.0013 | Indirectly inhibits downstream effects of IFN^[5]^ | Involved in viral immune evasion ^[6]^ |
| IFITM3 | HALLMARK_INTERFERON_ALPHA_RESPONSE | Up | 69.27 | 0.0019 | IFITM3 can promote IFN-α expression^[7]^ | 1. Antiviral defense ^[8]^ 2.Enhances innate immunity  Influences ^[9]^   3.Enhances adaptive immunity^[10]^ |
| B2M | HALLMARK_INTERFERON_ALPHA_RESPONSE | Up | 54.90 | 0.014 | No evidence suggests B2M directly activates or inhibits the IFN-α signaling pathway | 1.Promotes anti-tumor immunity^[11]^ 2.Promotes inflammation and oxidative stress^[12]^ 3.Represents a potential novel therapeutic target^[13]^ |
| LGALS3BP | HALLMARK_INTERFERON_ALPHA_RESPONSE | Up | 53.9452657833334 | 0.00229628929279369 | Activates (upregulates) ^[14]^ | 1.Modulates inflammation and immune response ^[15]^ 2.Antiviral immunity and innate immune enhancement^[16]^ |

**References**

[1] Gold IM, Reis N, Glaser F, Glickman MH. Coronaviral PLpro proteases and the immunomodulatory roles of conjugated versus free Interferon Stimulated Gene product-15 (ISG15). Semin Cell Dev Biol. 2022. 132: 16-26.

[2] Swaim CD, Scott AF, Canadeo LA, Huibregtse JM. Extracellular ISG15 Signals Cytokine Secretion through the LFA-1 Integrin Receptor. Mol Cell. 2017. 68(3): 581-590.e5.

[3] Sooryanarain H, Rogers AJ, Cao D, Haac M, Karpe YA, Meng XJ. ISG15 Modulates Type I Interferon Signaling and the Antiviral Response during Hepatitis E Virus Replication. J Virol. 2017. 91(19).

[4] Burks J, Fleury A, Livingston S, Smith JP. ISG15 pathway knockdown reverses pancreatic cancer cell transformation and decreases murine pancreatic tumor growth via downregulation of PDL-1 expression. Cancer Immunol Immunother. 2019. 68(12): 2029-2039.

[5] Zhang F, Attarilar S, Xie K, et al. Carfilzomib alleviated osteoporosis by targeting PSME1/2 to activate Wnt/β-catenin signaling. Mol Cell Endocrinol. 2022. 540: 111520.

[6] Liu Y, Yang J, Wang Y, et al. The proteasome activator subunit PSME1 promotes HBV replication by inhibiting the degradation of HBV core protein. Genes Dis. 2024. 11(6): 101142.

[7] Li C, Guo XR, Dong ZM, et al. Novel interacting proteins identified by tandem affinity purification and mass spectrometry associated with IFITM3 protein during PDCoV infection. Int J Biol Macromol. 2024. 277(Pt 2): 132755.

[8] Liu Y, Ma J, Gao P, et al. IFITM3 reduces infectious bursal disease virus proliferation by regulating interferon expression. Microb Pathog. 2024. 194: 106802.

[9] Wu R, Yang H, Liu C. IFIT3: a crucial mediator in innate immunity and tumor progression with therapeutic implications. Front Immunol. 2025. 16: 1515718.

[10] Bedford JG, O'Keeffe M, Reading PC, Wakim LM. Rapid interferon independent expression of IFITM3 following T cell activation protects cells from influenza virus infection. PLoS One. 2019. 14(1): e0210132.

[11] Zhao Y, Cao Y, Chen Y, et al. B2M gene expression shapes the immune landscape of lung adenocarcinoma and determines the response to immunotherapy. Immunology. 2021. 164(3): 507-523.

[12] Zhong Q, Zou Y, Liu H, et al. Toll-like receptor 4 deficiency ameliorates β2-microglobulin induced age-related cognition decline due to neuroinflammation in mice. Mol Brain. 2020. 13(1): 20.

[13] Ravindranath MH, Ravindranath NM, Amato-Menker CJ, Hilali FE, Filippone EJ. Conformational Alterations of the Cell Surface of Monomeric and Dimeric β2m-Free HLA-I (Proto-HLA) May Enable Novel Immune Functions in Health and Disease. Curr Issues Mol Biol. 2024. 46(7): 6961-6985.

[14] El Bannoudi H, Cornwell M, Luttrell-Williams E, et al. Platelet LGALS3BP as a Mediator of Myeloid Inflammation in Systemic Lupus Erythematosus. Arthritis Rheumatol. 2023. 75(5): 711-722.

[15] Sung M, Kim DH, Sun EG, et al. LGALS3BP Induces Insulin Resistance via TLR2-IKKα/β Pathway-Mediated IRS1 Serine Phosphorylation. Endocrinol Metab (Seoul). 2025 .

[16] Wang X, Zhang W, Zhang J, et al. Galectin 3-binding protein suppresses PRRSV replication via Cullin3-mediated ubiquitination degradation of non-structural protein 12. J Virol. 2025 : e0108325.
