# Supplementary material for: Manganese modulates hepatocellular carcinoma cytotoxicity and doxorubicin sensitivity in a dose dependent manner
Source: Front Oncol. 2026 Feb 13;16:1715702. doi: 10.3389/fonc.2026.1715702 (PMC12946836; doi:10.3389/fonc.2026.1715702)
Supplement: Supplementary file 13 [file Image1.pdf]

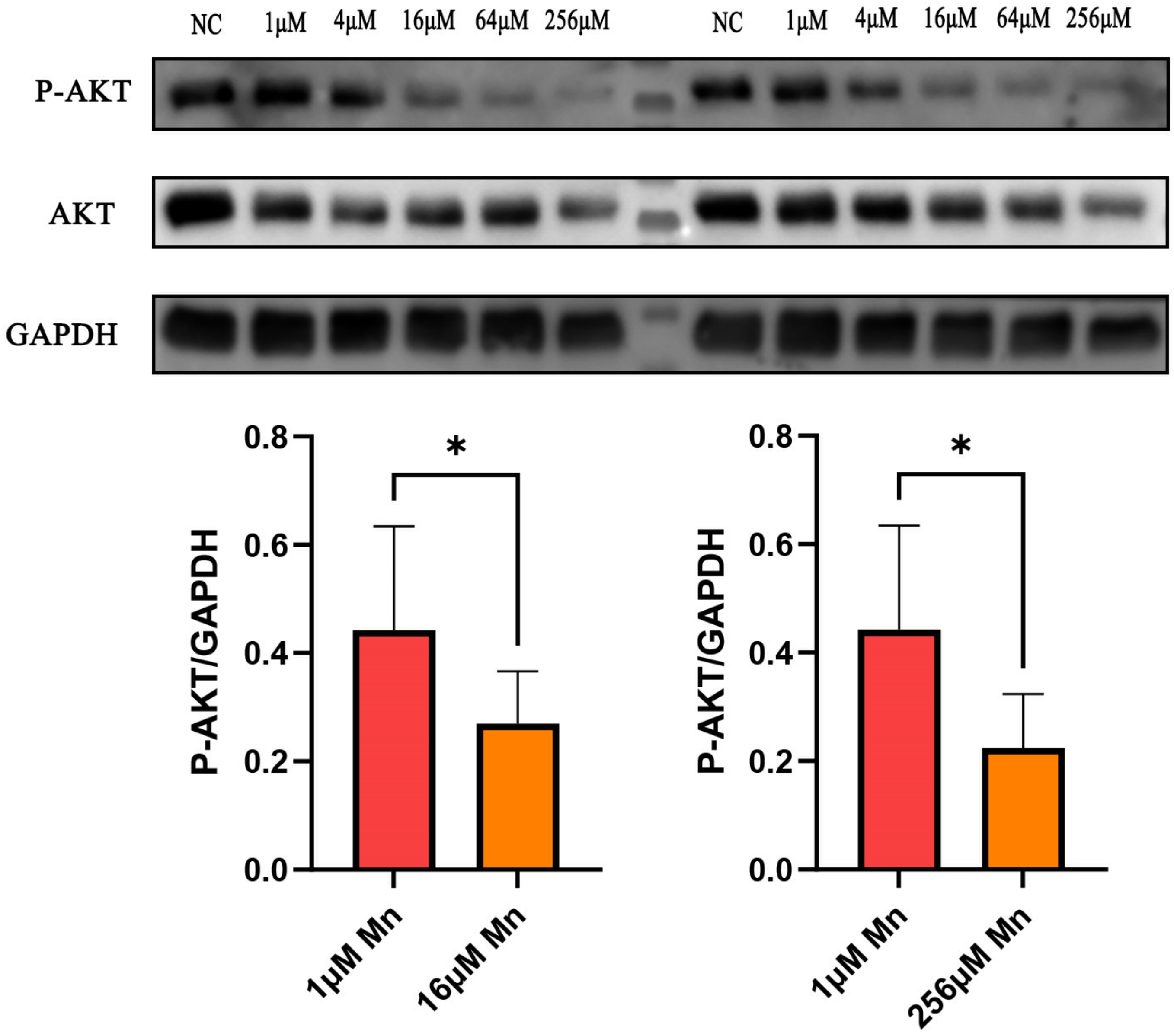

Supplementary Figure 5:
Western blot protein bands for AKT and phosphorylated AKT in Huh7 cells. The bands from left to right correspond to the negative control (NC) group, the 1μM manganese treatment group, the 4μM manganese treatment group, the 16 μM manganese treatment group, the 64μM manganese treatment group, and the 256μM manganese treatment group.
