# Supplementary material for: Manganese modulates hepatocellular carcinoma cytotoxicity and doxorubicin sensitivity in a dose dependent manner
Source: Front Oncol. 2026 Feb 13;16:1715702. doi: 10.3389/fonc.2026.1715702 (PMC12946836; doi:10.3389/fonc.2026.1715702)
Supplement: Supplementary file 14 [file Image2.pdf]

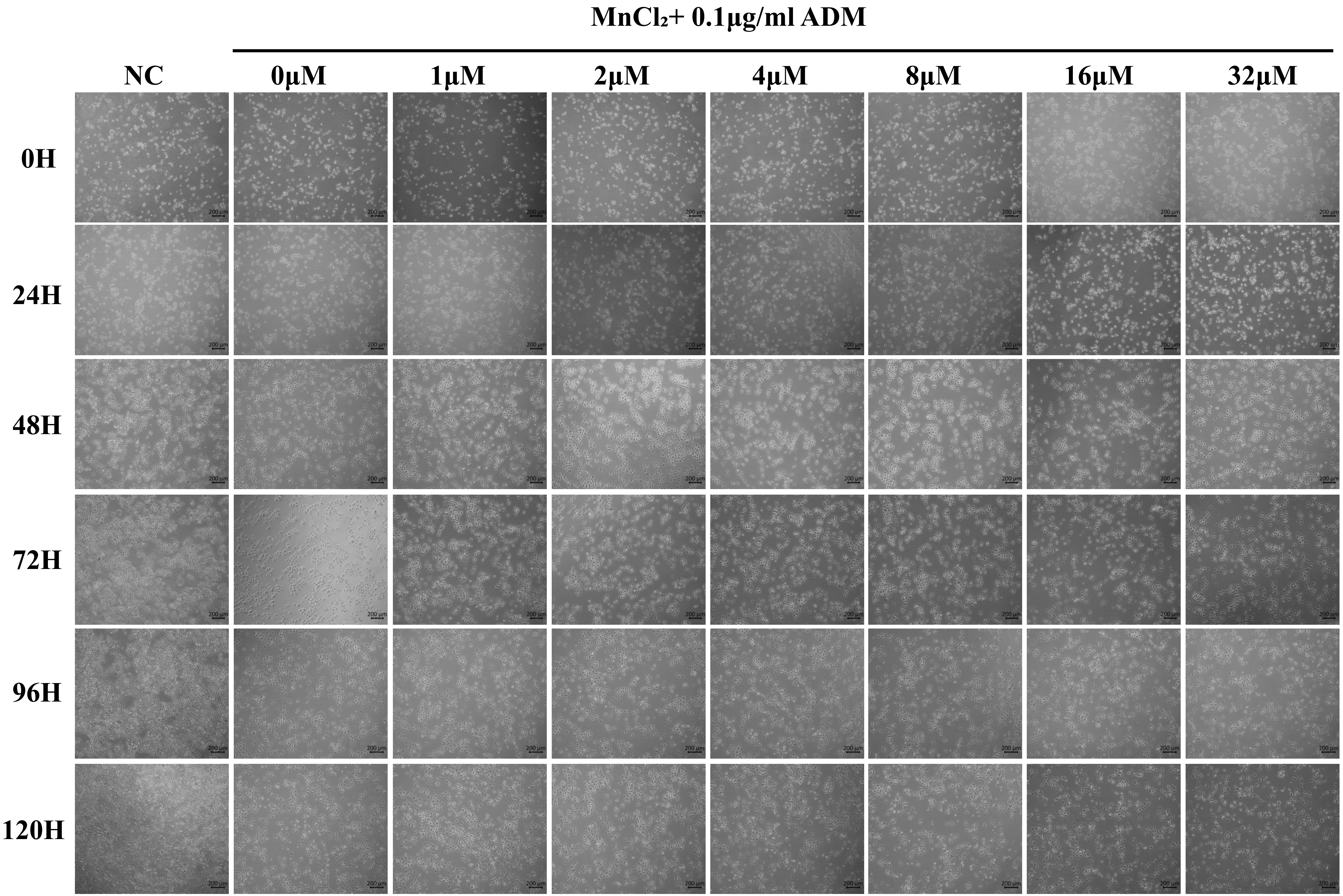

Supplementary Figure 7: Five-day Mn^{2+} co-treatment reveals morphology indicative of altered cell death mode (scale bar=200 μm).
