# Supplementary material for: Manganese modulates hepatocellular carcinoma cytotoxicity and doxorubicin sensitivity in a dose dependent manner
Source: Front Oncol. 2026 Feb 13;16:1715702. doi: 10.3389/fonc.2026.1715702 (PMC12946836; doi:10.3389/fonc.2026.1715702)
Supplement: Supplementary file 15 [file Image3.pdf]

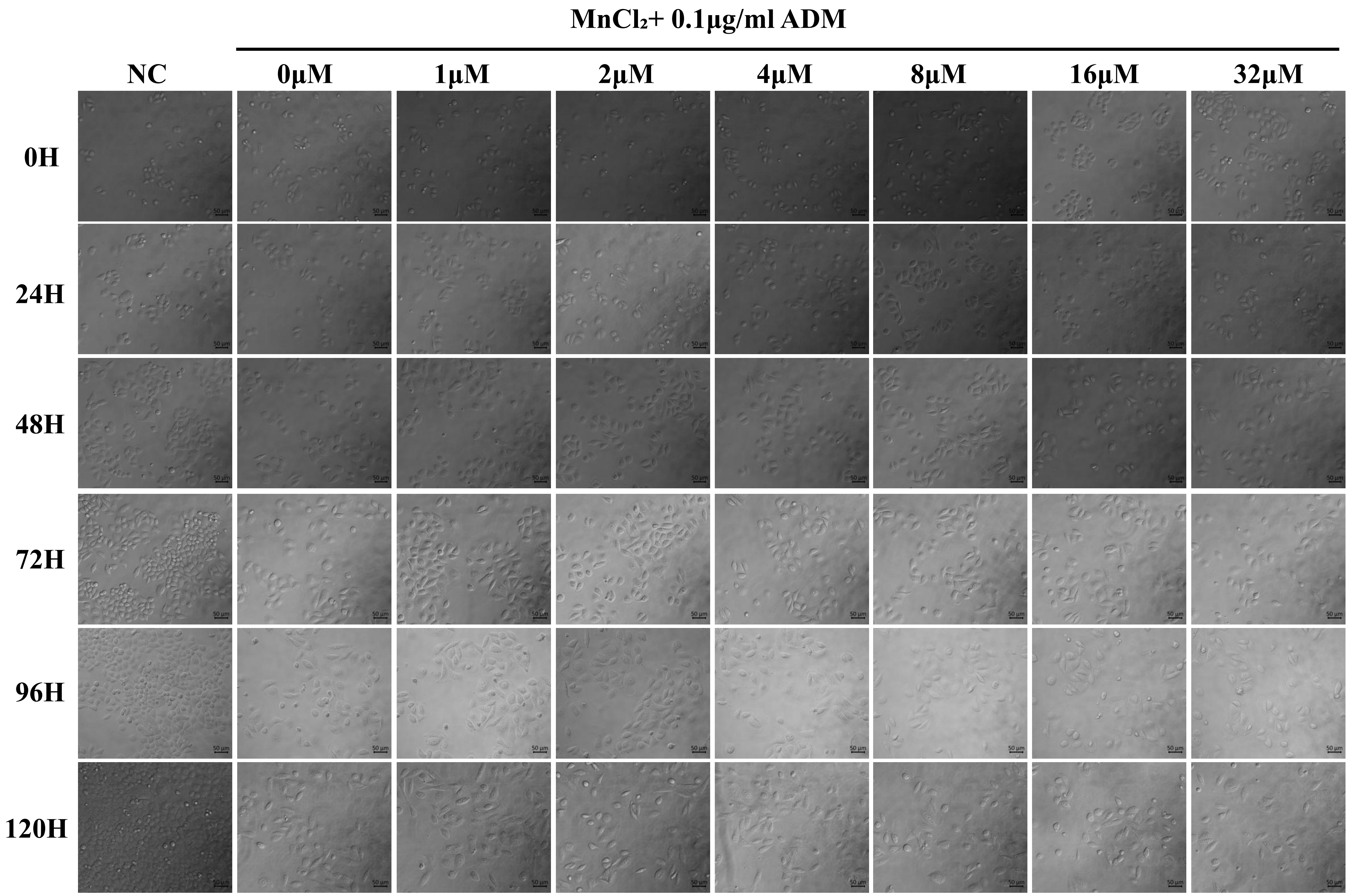

Supplementary Figure 8: Five-day Mn^{2+} co-treatment reveals morphology indicative of altered cell death mode (scale bar=50 μm).
