# Supplementary material for: Manganese modulates hepatocellular carcinoma cytotoxicity and doxorubicin sensitivity in a dose dependent manner
Source: Front Oncol. 2026 Feb 13;16:1715702. doi: 10.3389/fonc.2026.1715702 (PMC12946836; doi:10.3389/fonc.2026.1715702)
Supplement: Supplementary file 16 [file Image4.pdf]

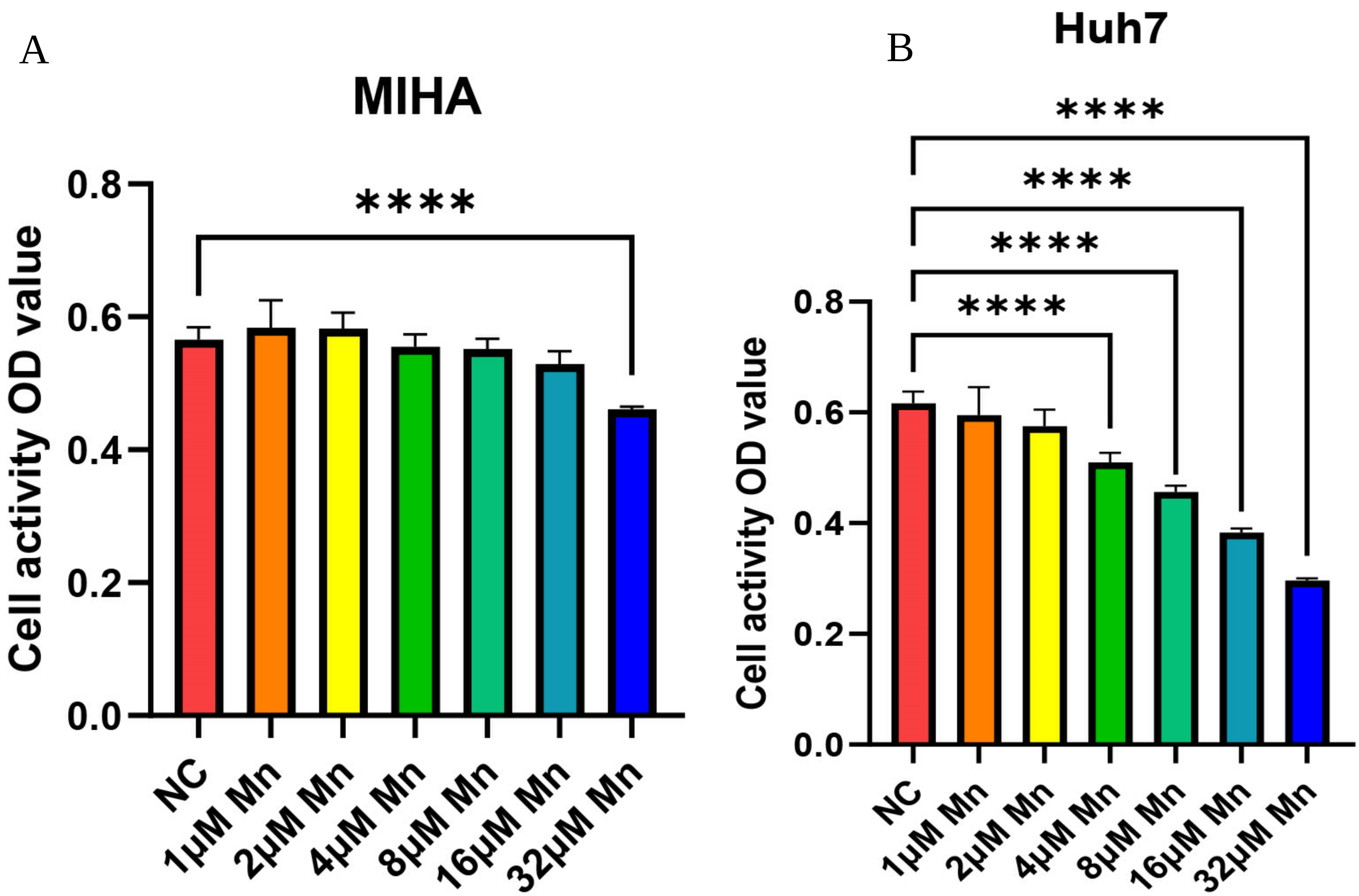

Supplementary Figure 4:
(a) Effects of different concentrations of manganese on MIHA cells. (b) Effects of different concentrations of manganese on Huh7 cells.
