# Supplementary figures and images for: Manganese modulates hepatocellular carcinoma cytotoxicity and doxorubicin sensitivity in a dose dependent manner
Source: Front Oncol. 2026 Feb 13;16:1715702. doi: 10.3389/fonc.2026.1715702 (PMC12946836; doi:10.3389/fonc.2026.1715702)

# Expression of ABCB1 and CPT1A

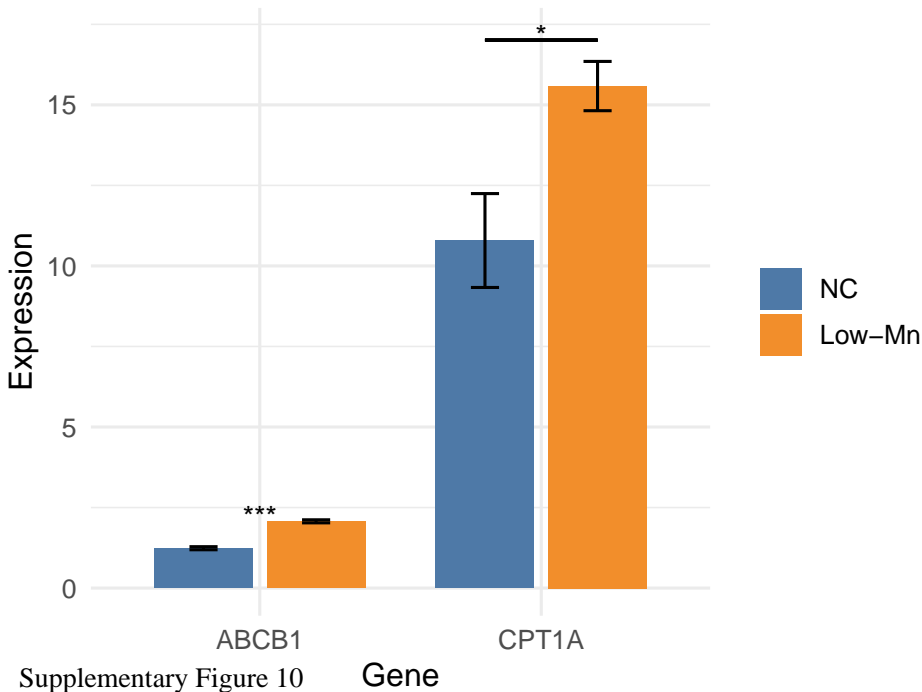

Supplement: Supplementary file 17 [file Image5.pdf]

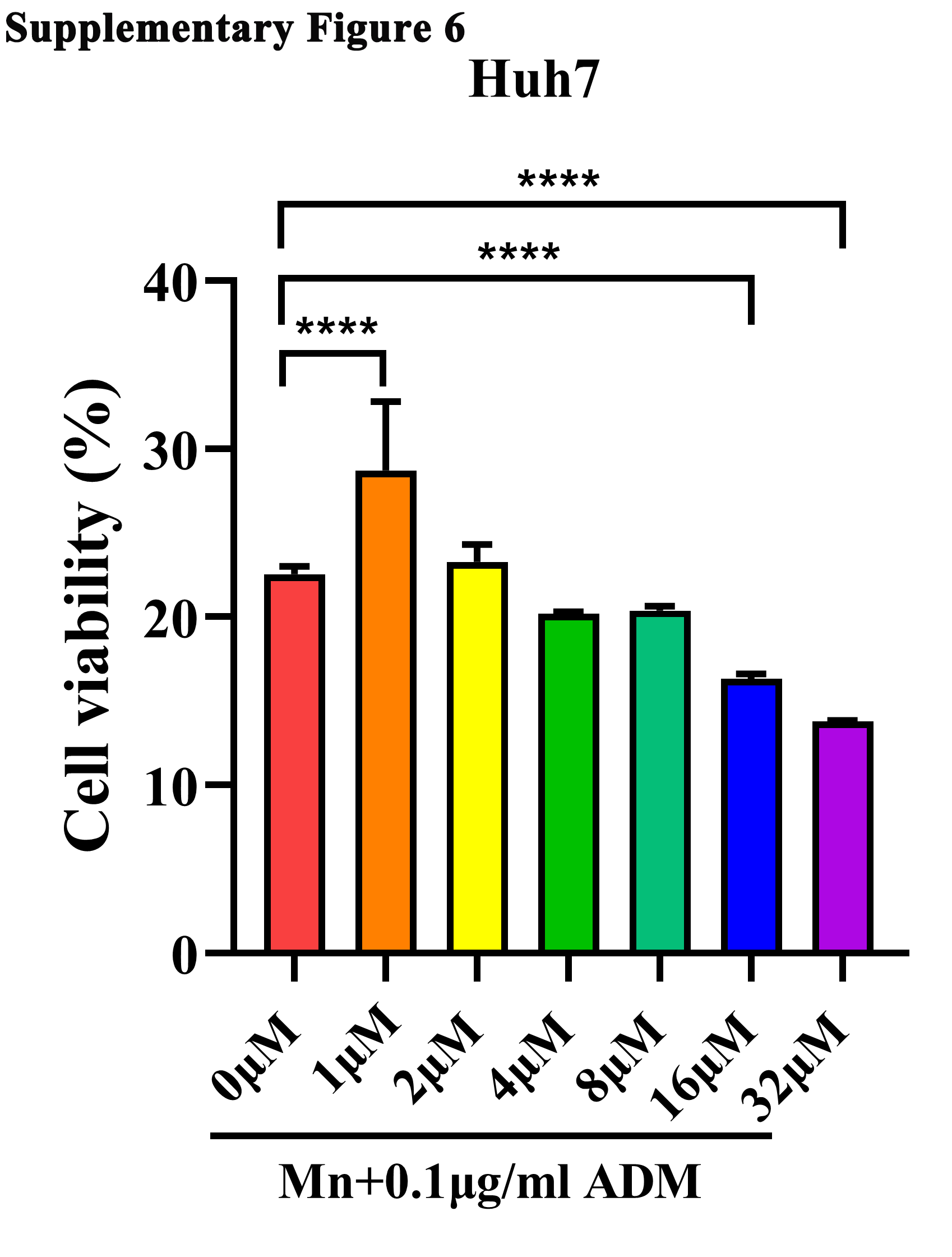

Supplement: Supplementary file 18 [file Image6.tif]

Supplementary Figure 9

## KEGG\_ABC\_TRANSPORTERS

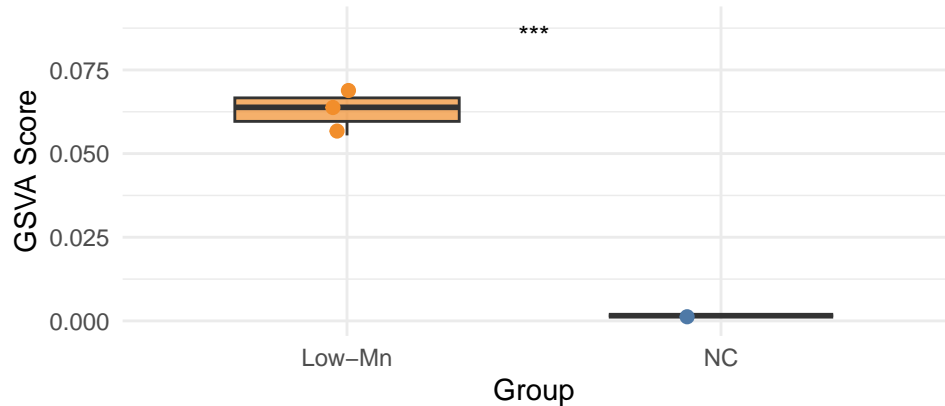

Supplement: Supplementary file 19 [file Image7.pdf]
